# Consequence of the tumor-associated conversion to cyclin D1b

**DOI:** 10.15252/emmm.201404242

**Published:** 2015-03-18

**Authors:** Michael A Augello, Lisa D Berman-Booty, Richard Carr, Akihiro Yoshida, Jeffry L Dean, Matthew J Schiewer, Felix Y Feng, Scott A Tomlins, Erhe Gao, Walter J Koch, Jeffrey L Benovic, John Alan Diehl, Karen E Knudsen

**Affiliations:** 1 Department of Cancer Biology, Thomas Jefferson UniversityPhiladelphia, PA, USA; 2 Kimmel Cancer Center, Thomas Jefferson UniversityPhiladelphia, PA, USA; 3 Department of Biochemistry and Molecular Biology, Thomas Jefferson UniversityPhiladelphia, PA, USA; 4 Medical University of South CarolinaCharleston, SC, USA; 5 Hollings Cancer CenterCharleston, SC, USA; 6 Michigan Center for Translational Pathology, University of Michigan Medical CenterAnn Arbor, MI, USA; 7 Department of Radiation Oncology, University of Michigan Medical CenterAnn Arbor, MI, USA; 8 Comprehensive Cancer Center, University of Michigan Medical CenterAnn Arbor, MI, USA; 9 Department of Urology, University of Michigan Medical CenterAnn Arbor, MI, USA; 10 Pharmacology & Center for Translational MedicinePhiladelphia, PA, USA; 11 Temple University School of MedicinePhiladelphia, PA, USA; 12 Department of Urology, Thomas Jefferson UniversityPhiladelphia, PA, USA; 13 Department of Radiation Oncology, Thomas Jefferson UniversityPhiladelphia, PA, USA

**Keywords:** cell cycle, cyclin, cyclin D1b, PARP

## Abstract

Clinical evidence suggests that cyclin D1b, a variant of cyclin D1, is associated with tumor progression and poor outcome. However, the underlying molecular basis was unknown. Here, novel models were created to generate a genetic switch from cyclin D1 to cyclin D1b. Extensive analyses uncovered overlapping but non-redundant functions of cyclin D1b compared to cyclin D1 on developmental phenotypes, and illustrated the importance of the transcriptional regulatory functions of cyclin D1b *in vivo*. Data obtained identify cyclin D1b as an oncogene, wherein cyclin D1b expression under the endogenous promoter induced cellular transformation and further cooperated with known oncogenes to promote tumor growth *in vivo*. Further molecular interrogation uncovered unexpected links between cyclin D1b and the DNA damage/PARP1 regulatory networks, which could be exploited to suppress cyclin D1b-driven tumors. Collectively, these data are the first to define the consequence of cyclin D1b expression on normal cellular function, present evidence for cyclin D1b as an oncogene, and provide pre-clinical evidence of effective methods to thwart growth of cells dependent upon this oncogenic variant.

## Introduction

D-Type cyclins link mitogenic stimuli to the cell cycle machinery and have well-established roles in cancer (Musgrove *et al*, 2011). Accumulation of cyclin D1 is enhanced in response to pro-proliferative signals and engages the cell cycle machinery by direct binding and activation of CDK4/6 complexes (Musgrove *et al*, 2011; Malumbres & Barbacid, 2009). cyclin D1/CDK4/6 subsequently phosphorylates key substrates (including the retinoblastoma tumor suppressor, RB) which induces progression from G1 to S phase (Malumbres & Barbacid, 2009). While these cell cycle regulatory functions of cyclin D1 have been well described and are conserved across most cell and tissue types (Musgrove *et al*, 2011; Malumbres & Barbacid, 2009), additional, largely CDK4/6-independent functions of cyclin D1 are known to be essential for distinct biological activities. The most well-described function of cyclin D1 outside cell cycle control is the ability to act as a transcriptional co-regulator (Bienvenu *et al*, 2010; Musgrove *et al*, 2011). The transcriptional regulatory activities of cyclin D1 have been shown in multiple systems to modulate pathways critical for both development and tumorigenesis (Bienvenu *et al*, 2010; Comstock *et al*, 2011, 2013; Musgrove *et al*, 2011; McMahon *et al*, 1999; Fu *et al*, 2005). Further insight was gleaned from analyses of the cyclin D1 interactome, which uncovered a large network of transcriptional regulators in complex with cyclin D1 (Bienvenu *et al*, 2010). Accordingly, parallel studies identified an essential domain of cyclin D1 that was responsible for steroid receptor interaction, termed the repressor domain (Petre-Draviam *et al*, 2003, 2005; Burd *et al*, 2005), which is paramount for modulation of steroid receptor action and associated downstream biology (Comstock *et al*, 2011, 2013; Zwijsen *et al*, 1997; Knudsen *et al*, 1999; Schiewer *et al*, 2009). Thus, the biological functions of cyclin D1 appear to be manifest through regulation of both cell cycle progression and selected gene expression networks.

Given the influential functions of cyclin D1 on cell proliferation and signaling, cyclin D1 expression is stringently regulated. In cancer cells, however, deregulation of cyclin D1 is common and is often associated with development and progression of human malignancies (Musgrove *et al*, 2011). Evidence from human tumors uncovered multiple mechanisms through which cyclin D1 expression is deregulated, most of which involve alterations of the *CCND1* locus itself (Musgrove *et al*, 2011). These include the following: (1) amplification of the *CCND1* gene (observed frequently in breast, pancreatic, skin, lung, and head and neck cancers) (Arnold & Papanikolaou, 2005; Garcea *et al*, 2005; Thomas *et al*, 2005; Li *et al*, 2006; Gautschi *et al*, 2007), (2) translocation of the *CCND1* locus to *IGH* enhancer elements (seen in nearly all cases of mantle cell lymphoma) (Bosch *et al*, 1994; Bigoni *et al*, 1996), and (3) mutations in the sequences encoding the cyclin D1 PEST domain (which occur in esophageal cancers) (Benzeno *et al*, 2006). While these genetic aberrations are associated with altered cyclin D1 expression, the clinical significance has proven to be highly variable. In mantle cell lymphoma (MCL), deregulation of the *CCND1* locus is prognostic and has been implicated as a driver of disease onset, but is dispensable for disease progression (Bertoni *et al*, 2006; Gladden *et al*, 2006). Among other malignancies with cyclin D1 locus alterations, there is little evidence of association with tumor progression. For example, no consistent association of cyclin D1 amplification was found with response to therapeutic challenge in patients with hormone receptor-positive breast cancer (Lundgren *et al*, 2012; Rudas *et al*, 2008). Furthermore, mutations within the coding region of cyclin D1, found in cases of esophageal and endometrial cancer (Moreno-Bueno *et al*, 2003; Benzeno *et al*, 2006), have yet to be associated with clinical markers of disease progression, together suggesting that the observed oncogenic functions of cyclin D1 involve additional mechanisms of deregulation. Consistent with this concept, emerging clinical evidence suggests that alternative splicing of cyclin D1 transcript (cyclin D1a) to the shorter isoform cyclin D1b occurs frequently in human malignancy (Augello *et al*, 2014; Musgrove *et al*, 2011; Wang *et al*, 2008; Comstock *et al*, 2009), and appears to be a major mechanism though which cyclin D1 exerts its oncogenic activity.

Cyclin D1b represents a putatively neoplastic-specific isoform of cyclin D1, which arises as a failure to splice at the exon 4/intron 4 boundary of the cyclin D1 pre-mRNA (Solomon *et al*, 2003; Knudsen, 2006). This results in the incorporation of an early stop codon and loss of C-terminal-encoded sequences important for the transcriptional regulatory functions and stability of full-length cyclin D1 (termed cyclin D1a). Consequently, cyclin D1b harbors a unique 33 amino acid C-terminus, which represents a ‘gain-of-function’ cyclin D1 variant (Augello *et al*, 2014; Lu *et al*, 2003; Solomon *et al*, 2003). This concept is supported by clinical evidence demonstrating that cyclin D1b is induced in lymphoma, esophageal, breast, lung, and prostate cancer (Augello *et al*, 2014; Lu *et al*, 2003; Carrere *et al*, 2005; Li *et al*, 2008; Comstock *et al*, 2009; Millar *et al*, 2009). Furthermore, in contrast to cyclin D1a, cyclin D1b expression is associated with tumor progression and therapeutic failure in breast (Wang *et al*, 2008) and prostate cancer (Augello *et al*, 2014; Comstock *et al*, 2009), and is an independent predictor of poor prognosis and survival in small-cell lung cancer (Li *et al*, 2008). Although these findings suggest that cyclin D1b represents an oncogenic isoform of cyclin D1, the molecular impact of the endogenous switch to cyclin D1b, as occurs in tumors, remains unclear.

Given the established clinical importance of cyclin D1b induction, novel models harboring a genetic switch from cyclin D1a to cyclin D1b were generated herein and uncovered the effects of the switch to cyclin D1b expression on developmental and cellular transformation phenotypes. First, extensive *in vivo* analyses uncovered overlapping but non-redundant functions with that of cyclin D1a, providing the first evidence of divergent action of this isoform on normal cellular processes. Furthermore, *in vitro* models provided evidence to support the role of cyclin D1b as an oncogene, fostering transformation of primary cells and cooperating with established oncogenes to drive tumor formation *in vivo*. Importantly, cyclin D1b-mediated transformation was associated with enhanced presence of markers of DNA damage, providing mechanistic insight into the oncogenic action of this isoform. This is of potential clinical relevance, as cyclin D1b-transformed cells were hypersensitized to cell cycle arrest and senescence through combined genotoxic insult, providing preclinical insight into mechanisms that can be utilized to target cyclin D1b-expressing tumors. This study thus established a first-in-field model of the switch to cyclin D1b, identified unique roles for this isoform, and put forth novel strategies for treating cyclin D1b-positive tumors.

## Results

### Humanization of the *Ccnd1* exon 4/5 locus results in exclusive production of cyclin D1b

**Figure 1:**
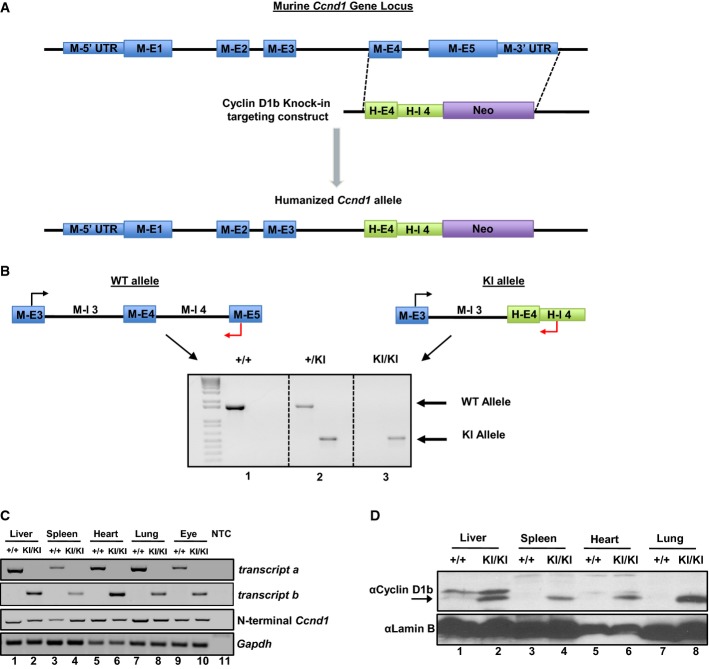
Humanization of the *Ccnd1* exon 4/5 locus results in exclusive production of cyclin D1b

To develop robust genetic systems of cyclin D1b production under the endogenous promoter, a gene-targeting construct was generated wherein all C-terminal-encoding components of the murine *Ccnd1* gene were replaced with the C-terminal sequences responsible for human cyclin D1b production. As shown in Fig1A, this was accomplished by replacing murine exon 4, intron 4, exon 5, and 3′ UTR with human exon 4 and intron 4 encoding sequences. The use of human exon 4/intron 4 and removal of murine exon 5/3′ UTR were necessary to both eliminate the possibility of full-length *transcript a* production (encoding cyclin D1a), and to foster production of *transcript b*, encoding the unique C-terminus harbored by cyclin D1b. Furthermore, this strategy preserves upstream splicing events of the *Ccnd1* transcript, which more accurately reflects the biochemical conditions responsible for cyclin D1b production.

Representative schematic of the targeting construct generated to humanize the *Ccnd1* exon 4/5 genomic locus to produce cyclin D1b.

Top: Schematic of primer pairs designed to discriminate between wild-type and knock-in alleles. Bottom: Representative genotyping of *Ccnd1*^+/+^*, Ccnd1*^+/^^*KI*^, and *Ccnd1*^*KI*^^*/*^^*KI*^ mice validating specificity of the primer pairs and somatic insertion of the targeting construct.

PCR analysis of *transcript b* expression in organs harvested from *Ccnd1*^+/+^ and *Ccnd1*^*KI*^^*/*^^*KI*^ mice, demonstrating production of *transcript b* specifically in KI mice. *Gapdh* serves as a control (NTC, Non-template control).

Immunoblot from parallel samples in (C) utilizing antisera specific to the 33 amino acids generated by human *CCND1* intron 4. Lamin B serves as a control. Arrow indicates the cyclin D1b band.

Generation of cyclin D1b knock-in mice was accomplished through electroporation of the targeting knock-in construct (Fig1A) into murine embryonic stem cells. Heterozygous clones were identified by Southern blot analysis and injected into developing mouse blastocysts, generating chimeric mice. Chimeric mouse pairs were subsequently bred to produce heterozygous wild-type/cyclin D1b mice (here-to-after referred to as ‘+’ and ‘KI’ alleles respectively), which were then crossed to produce homozygous cyclin D1b knock-in mice (*Ccnd1*^*KI/KI*^). Primers specific for murine exon 5 (‘+’ allele) or human intron 4 (‘KI’ allele) were used to distinguish between the respective genotypes (Fig1B, top), and confirm somatic incorporation of the KI allele (Fig1B, bottom). To verify that humanization of the *Ccnd1* locus resulted in the production of *transcript b*, individual tissues previously reported to express cyclin D1 were harvested from *Ccnd1*^+/+^ and *Ccnd1*^*KI/KI*^ mice and analyzed for cyclin D1 expression. Primer pairs specific to the N-terminus of cyclin D1 (common to both *transcript a* and *b*) were used to confirm expression in each tissue by PCR (Fig1C). Further investigation using unique primer sets validated exclusive production of *transcript b* in *Ccnd1*^*KI/KI*^ animals, and expression was mirrored at the protein level in all tissue types tested (Fig1D), affirming that humanization of the *Ccnd1* locus results in the exclusive production of cyclin D1b. Thus, this system provides a unique tool to study cyclin D1b function under the control of its endogenous promoter and in the genetic absence of cyclin D1a.

### Unique functions of cyclin D1b in development

#### Ccnd1^KI/KI^ mice exhibit post-natal growth retardation

**Figure 2:**
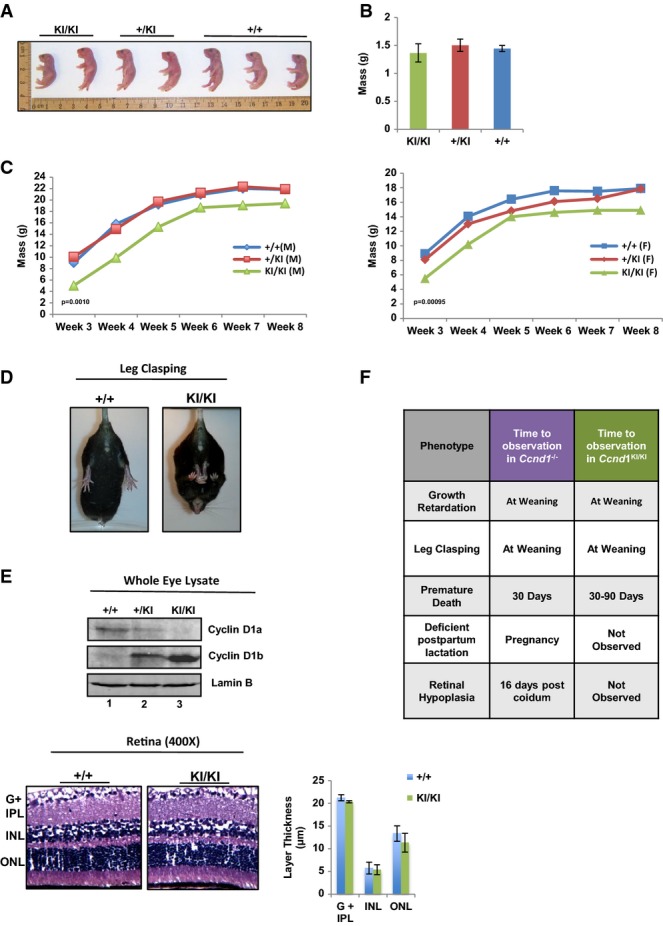
Cyclin D1b selectively rescues *Ccnd1*^−/−^ phenotypes

While several murine models have been characterized which mutate and/or toggle cyclin D1 expression, to date no genetic systems had been generated which assess cyclin D1b function under the endogenous promoter *in vivo*. Crosses between *Ccnd1*^*+/KI*^ mice (> 20 mating pairs across multiple generations) revealed that *Ccnd1*^*KI/KI*^ mice are born in typical Mendelian ratios (Supplementary Fig S1A), suggesting that cyclin D1b expression does not result in embryonic lethality. At birth, *Ccnd1*^*KI/KI*^ pups were indistinguishable from wild-type littermates, as noted by virtually identical size (Fig2A) and mass (Fig2B). However, by 3 weeks of age, a significant reduction in size and weight was noted in the *Ccnd1*^*KI/KI*^ mice, which persisted over a period of 8 weeks and was independent of gender (Fig2C). Further analysis of individual organ weight (adjusted for total body mass) revealed no significant difference between *Ccnd1*^+/+^, *Ccnd1*^*+/KI*^, or *Ccnd1*^*KI/KI*^ animals, suggesting that diminished organ size was not causative for the observed reduction in mass. Notably, the growth rate of all animals was similar between 3 and 8 weeks of age, indicating that the reduction in size and mass occurs early in post-natal development. Interestingly, previous work modeling cyclin D1 loss (*Ccnd1*^−/−^) in an identical genetic background found a similar growth phenotype during early development, which persisted throughout the lifetime of *Ccnd1*^−/−^ animals (Sicinski *et al*, 1995). Given the similarity between these two models, these data support the concept that cyclin D1b induction is not sufficient to restore the growth retardation phenotype observed in *Ccnd1*^−/−^ mice and highlights the functional differences between the two cyclin D1 isoforms.

Neonates from 2 independent litters of *Ccnd1*^+/*KI*^ × *Ccnd1*^+/^^*KI*^ crosses were sacrificed at birth and genotyped. Mice were organized by genotype and total size measured.

Mass of neonates was calculated at birth and average mass quantified (*n *= at least 4 mice per group).

Mice were weighed weekly, and growth is plotted as the average mass of each genotype/week (left: male, right: female, *n *>* *5 per gender and genotype).

Mice were held by the tails and analyzed for the leg clasping phenotype. Representative images of *Ccnd1*^+/+^ and *Ccnd1*^*KI*^^*/*^^*K*I^ age-matched mice are shown (*n *>* *10 for each group).

Top: Individual whole-cell lysates were generated from the eyes of mice of each genotype and analyzed for expression of +/+ cyclin D1 (cyclin D1a) and KI/KI cyclin D1b via immunoblot using antisera specific to each isoform. Bottom: Representative H&E staining of retinal tissue from *Ccnd1*^+/+^ and *Ccnd1*^*KI*^^*/*^^*KI*^ mice showing individual layers of the retina. The ganglion cell layer and inner plexiform layer (G + IPL), inner nuclear layer (INL), and outer nuclear layer (ONL) were quantified (right). Images were taken at 400× magnification.

Comparison of the phenotypes described in the *Ccnd1*^−/−^ mouse with those observed in the *Ccnd1*^*KI*^^*/*^^*KI*^ mouse, previously described (Sicinski *et al*, 1995). Data information: *n *= at least 4 for each genotype. Error bars represent the standard error of the mean (SEM), and significance was determined using a two-tailed Student's *t*-test.

#### Ccnd^KI/KI^ mice phenocopy neuromuscular and death phenotypes of the *Ccnd1*^−/−^ mouse

The latent growth phenotypes common between the *Ccnd1*^*KI/KI*^ and the *Ccnd1*^−/−^ mice (on identical genetic backgrounds) suggest the presence of overlapping but non-redundant functions of cyclin D1b and cyclin D1a. To further explore this concept, *Ccnd1*^*KI/KI*^ mice were initially evaluated for the presence of a neuromuscular hindlimb abnormality (leg clasping), which occurs with high frequency in mice that harbor cyclin D1 loss but is not present in *Ccnd1*^+/+^ or *Ccnd1*^+/−^ animals. Consistent with the growth phenotypes described above, this phenotype was observed in all *Ccnd1*^*KI/KI*^ mice examined (Fig2D, right) and was 100% penetrant across generations. While the severity with which *Ccnd1*^*KI/KI*^ animals clasp limbs varied (with the smallest animals tending to present with the most severe clasping), it was evident in all *Ccnd1*^*KI/KI*^ mice beginning at 3 weeks of age and persisted.

While both *Ccnd1*^*KI/KI*^ and *Ccnd1*^−/−^ mice display leg clasping and latent growth phenotypes in early development, notable differences become apparent through aging. *Ccnd1*^−/−^ animals show minor frequency of spontaneous morbidity beginning at 3 weeks (Sicinski *et al*, 1995), observable up to 2 months of age. This phenotype was also apparent in *Ccnd1*^*KI/KI*^ animals beginning at 1 month, the sudden death phenotype persisted up to 3 months, with no preceding signs of lethargy, malnutrition, or dehydration (Supplementary Fig S1A). Animals that survived beyond 3 months did not succumb to sudden death. Initial tests were undertaken to determine whether critical organ functions were compromised in *Ccnd1*^*KI/KI*^ mice. Analyses of complete blood counts and serum biochemistry analyses showed no evidence of hematopoietic, liver, pancreatic, or kidney dysfunction in either *Ccnd1*^+/+^ or *Ccnd1*^*KI/KI*^ mice. Accordingly, no clinically significant abnormalities were histologically apparent in mice aged to 9 months in the heart, liver, lung, brain, spleen, small intestine, colon, kidney, adrenals, skin, bone/bone marrow, urinary bladder, stomach, testes, uterus, thyroid, or thymus of either the *Ccnd1*^*KI/KI*^ or *Ccnd1*^+/+^ animals. Analysis of cardiac function utilizing echocardiogram revealed no major defect in overall cardiac output (Supplementary Fig S2A) between genotypes, but echo-derived mass and thickness of the left ventricle were consistently diminished in *Ccnd1*^*KI/KI*^ animals (Supplementary Fig S2B) suggesting a potential role for cyclin D1/1b in this tissue type. Collectively, these data suggest the low frequency of sudden death observed in *Ccnd1*^*KI/KI*^ mice is complex, and further highlight the divergent action of cyclin D1b on developmental phenotypes.

#### Ccnd1^KI/KI^ mice exhibit normal mammary and retinal development

Female mice lacking cyclin D1 expression are unable to effectively lactate in response to pregnancy (Sicinski *et al*, 1995), resulting in neonatal death. Consonantally, litters born of *Ccnd1*^*KI/KI*^ mothers exhibit a similar death response within 1 week of birth (notably, not seen with *Ccnd1*^*+/KI*^ mothers). This was not attributable to birth defects, as this phenotype is readily reversed by replacing *Ccnd1*^*KI/KI*^ females with *Ccnd1*^*KI/+*^ or *Ccnd1*^+/+^ foster mothers after birth (Supplementary Fig S1B). Interestingly, this was not due to lack of mammary gland development in *Ccnd1*^*KI/KI*^*; a*nalysis of mammary gland branching and histoarchitecture post-pregnancy uncovered no appreciable differences between *Ccnd1*^*KI/KI*^ or *Ccnd1*^+/+^ females (Supplementary Fig S1C). This suggests that the observed neonatal death from *Ccnd1*^*KI/KI*^ mothers is not due to deficient mammary growth and that other (potentially neurological) behaviors likely underlie such phenotypes.

Of all the phenotypes associated with *Ccnd1*^−/−^ mice, the most penetrant and dramatic is improper development of the retina. In *Ccnd1*^−/−^ mice, layers of the retina fail to develop fully, resulting in retinal hypoplasia and limited visual capacity (Sicinski *et al*, 1995). Thus, to define the consequence of the genetic switch to cyclin D1b on retinal phenotypes, expression of individual cyclin D1 isoforms was initially characterized in whole-eye lysates. As shown in Fig2E, top, both cyclin D1a (lanes 1, 2) and cyclin D1b (lanes 2, 3) were readily detected. Furthermore, hematoxylin and eosin (H&E) staining of retinal tissue from aged-matched *Ccnd1*^*KI/KI*^ mice showed that the size of the individual retinal layers in *Ccnd1*^*KI/KI*^ mice is indistinguishable from that in *Ccnd1*^+/+^ (Fig2E bottom, quantified to the left). This is in contrast to *Ccnd1*^−/−^ mice, where dramatic hypoplasia of retinal layers was confirmed (Supplementary Fig S1D). Thus, whereas cyclin D1b insufficiently rescues the leg clasping and early sudden death phenotypes associated with cyclin D1a loss, the retinal phenotype is completely rescued by conversion to cyclin D1b. These collective data suggest that cyclin D1b harbors over-lapping but non-redundant functions with cyclin D1a (Fig2F), and reinforces the concept that cyclin D1b holds unique functions that impinge upon development.

### Genetic evidence for cyclin D1b as a *bona fide* oncogene

**Figure 3:**
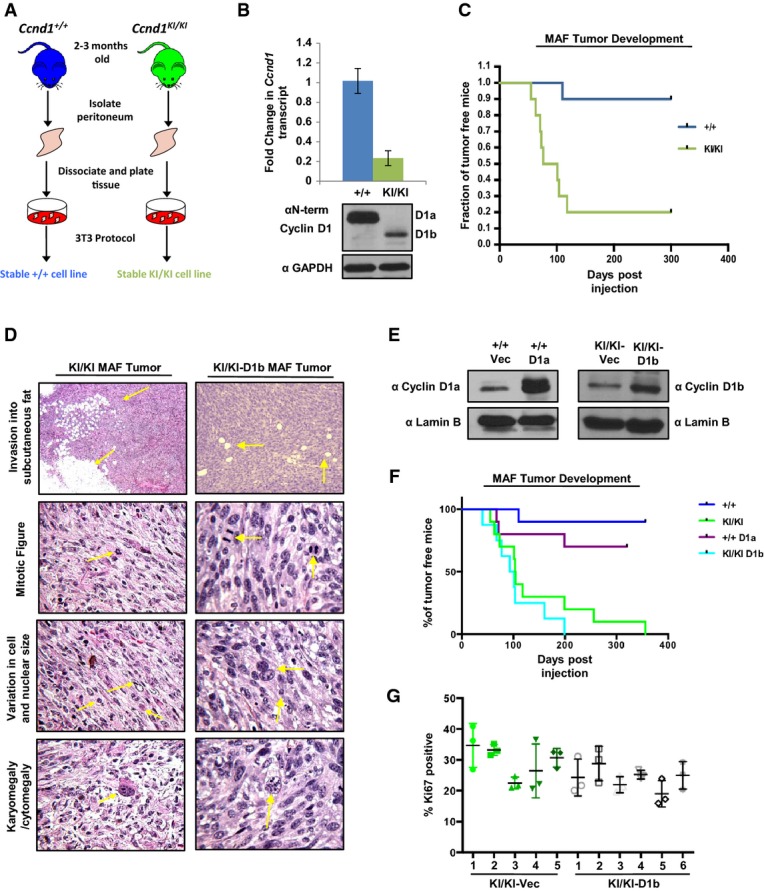
Genetic evidence for cyclin D1b as a credentialed oncogene

The model of endogenous cyclin D1b characterized above revealed unique contributions of cyclin D1b to developmental processes and illuminated the first evidence of biological distinctions between the two isoforms. In human cancer, high cyclin D1b is associated with the poor outcome (Li *et al*, 2008; Millar *et al*, 2009), and cyclin D1b overexpression transforms cells of mesenchymal origin (Lu *et al*, 2003). However, the oncogenic capacity of cyclin D1b in the absence of endogenous cyclin D1a or via the endogenous *CCND1* promoter had not been demonstrated. Murine models of forced cyclin D1a overexpression show limited oncogenic capacity (Wang *et al*, 1994); moreover, tumor development is not fully penetrant and is quite latent. Similar results were observed herein utilizing models of cyclin D1b expression driven from the endogenous promoter. *Ccnd1*^*KI/KI*^ mice up to 9 months of age showed no significant difference in the number hyperplastic or neoplastic lesions compared to age-matched wild-type controls, though animals are currently being aged further to quantify tumor formation rates. To expedite generation time and define the potential of an endogenous shift to cyclin D1b on tumor formation, murine adult fibroblasts (MAFs) were generated from the peritoneum of *Ccnd1*^+/+^ and *Ccnd1*^*KI/KI*^ mice (Fig3A). Similar models that modulated the expression of various oncogenes and tumor suppressors have demonstrated that MAF lines are an effective tool with which to study both biochemical and transformation phenotypes (de Napoles *et al*, 2004; Powers *et al*, 2004; Dean *et al*, 2010; Bourgo *et al*, 2011) and as such, each line was assayed for evidence of cellular transformation. Exclusive expression of respective cyclin D1 isoforms was initially confirmed by immunoblot using an antibody common to both cyclin D1a and cyclin D1b (Fig3B). Individual *Ccnd1*^+/+^ and *Ccnd1*^*KI/KI*^ lines were mixed with Matrigel and injected subcutaneously into the flanks of nude mice. As is shown in Fig3C, *Ccnd1*^+/+^ control cells were non-tumorigenic. By contrast, *Ccnd1*^*KI/KI*^ cells formed tumors by ~15 weeks with high penetrance (80%). H&E staining confirmed the tumors were of mesenchymal origin, and displayed hallmarks of neoplasia including (1) invasion into the subcutaneous fat, (2) nuclear and cytoplasmic atypia, and (3) prevalence of high mitotic figures (Fig3D, left). These data are the first to demonstrate that physiological levels of cyclin D1b are sufficient to promote tumorigenesis in immortalized cells, and provide evidence to support its role as an oncogene.

Murine adult fibroblasts (MAFs) were harvested from the peritoneum of *Ccnd1*^+/+^ and *Ccnd1*^*KI*^^*/*^^*KI*^ mice, and stable cell lines were generated utilizing a 3T3 protocol.

Indicated passage-matched MAF lines were grown to 70% confluency and then harvested for RNA and protein extraction. Top: qPCR analysis of cyclin D1 transcript using primer pairs common to both transcript a and transcript b. Bottom: Immunoblot of cyclin D1 levels in *Ccnd1*^+/+^ and *Ccnd1*^*KI*^^*/*K^MAF lines using an antibody common to both isoforms.

Percentage of tumor-free mice post-injection of 1 million cells mixed with Matrigel (1:1 ratio) into the flanks of nude mice over a period of 10 months (*n *=* *10 per genotype).

Indicated tumors were harvested 3 weeks after detectable tumor formation, fixed in formalin, and stained with H&E. Tumors were analyzed for features of malignancy by a board-certified veterinary pathologist. Arrows indicate the specific tumor-associated features noted. Top panels were taken at 40× magnification, and lower panels were taken at 400× magnification.

*Ccnd1*^+/+^ or *Ccnd1*^*KI*^^*/*^^*KI*^ MAF lines were stably transfected with either cyclin D1a (+/+), cyclin D1b (KI/KI) or vector control constructs. After selection with puromycin, individual lines were assessed for the induction of cyclin D1a in the +/+ line and cyclin D1b in KI/KI lines via immunoblot.

One million cells from the indicated stable cell lines were injected subcutaneously into the flanks of nude mice and monitored for tumor formation over a period of 365 days. Indicated time points represent time of palpable tumor detection and are plotted as % of tumor-free mice over time.

Sections from KI/KI vec and KI/KI-D1b tumors were stained for the proliferative marker Ki67. Three random fields from each tumor were quantified for Ki67 positivity, and are plotted as the mean ratio of Ki67-positive cells/total cell number for each individual tumor. Data information: Error bars represent ± SEM.

Notably, characterization of cyclin D1 levels in both the *Ccnd1*^+/+^ and *Ccnd1*^*KI/KI*^ lines uncovered reduced expression of cyclin D1b compared to cyclin D1a at both the protein and transcript level (Fig3B). Since elevated D-type cyclins have been associated with tumor formation and genome instability *in vivo* (Wang *et al*, 1994; Casimiro *et al*, 2012), the effect of elevated levels of cyclin D1a and cyclin D1b was next interrogated for tumor kinetics *in vivo*. Isogenic cell lines were engineered to overexpress either cyclin D1a (in *Ccnd1*^+/+^ MAFs +/+-D1a) or cyclin D1b (in *Ccnd1*^*KI/KI*^ MAFs- KI/KI-D1b) (Fig3E), subcutaneously implanted into the flanks of nude mice, and monitored for tumor growth over a period of 1 year. Consistent with previous data, the induction of cyclin D1a in *Ccnd1*^+/+^ lines was weakly tumorigenic, increasing tumor formation of that cell type by ~20% (Fig3F). Importantly, there was no observable increase in the tumor kinetics or frequency between vector control and cyclin D1b-overexpressing *Ccnd1*^*KI/KI*^ lines. Analysis of pathological hallmarks of transformation in KI/KI-D1b lines found similar characteristics to that of the vector control (Fig3D, right), and no difference in proliferative capacity (as measured by Ki67) (Fig3G). Taken together, these data demonstrate that the presence of cyclin D1b can promote transformation and tumor formation and that elevated levels of this isoform are not required for its oncogenic activity.

**Figure 4:**
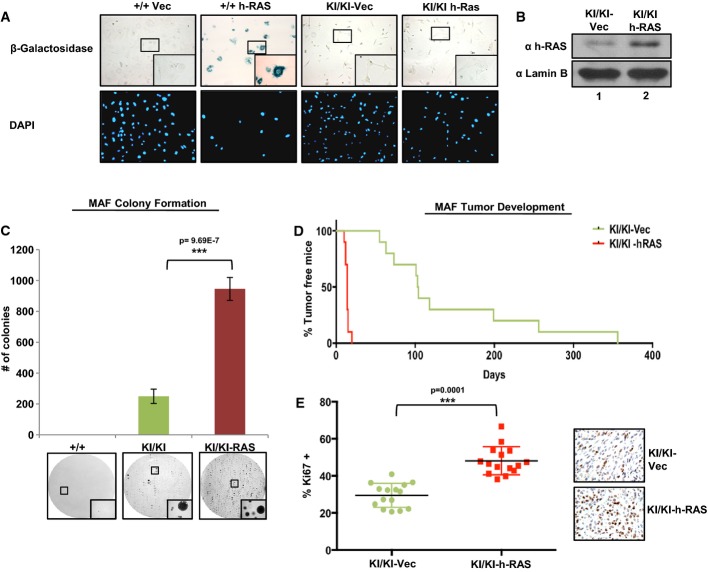
Cyclin D1b cooperates with h-Ras to drive tumorigenesis

Previous studies have suggested that mammary transformation and tumor development are critically dependent upon the kinase activity of cyclin D1 (Landis *et al*, 2006), whereby knock-in of a kinase dead allele of cyclin D1 (K112E) dramatically inhibited tumor formation within this tissue type (Landis *et al*, 2006). To define the ability of cyclin D1b to cooperate with oncogenes reliant on the cyclin D1/CDK4 pathway, lentivirus containing vector- or h-RAS-expressing constructs was introduced to both *Ccnd1*^+/+^ (+/+ h-Ras) and *Ccnd1*^*KI/KI*^ (KI/KI h-Ras) lines. While no anti-proliferative effects were noted in KI/KI h-Ras lines, +/+ h-Ras lines underwent sustained cell cycle arrest. Consistent with this observation, analysis of senescence markers (β-galactosidase activity) uncovered intense positive blue staining only in the +/+ h-Ras line (+/+ vec lines were negative) (Fig4A), demonstrating that in this cell type, h-Ras induction promotes oncogene-induced senescence, consistent with previous findings (Weyemi *et al*, 2012; Peeper *et al*, 2002). Given that KI/KI h-Ras lines were refractory to this effect (Fig4B), +/+ Vec, KI/KI vec, and KI/KI h-Ras lines were assayed their ability to form colonies in soft agar, a known marker of transformation. Consistent with tumor formation *in vivo* (Fig3A), +/+ *vec* lines were unable to grow in soft agar, while KI/KI vec cells were capable of forming colonies after 4 weeks (Fig4C). Strikingly, the addition of h-Ras enhanced the ability of these cells to grow in soft agar, increasing colony number by ~fivefold. This effect was further demonstrated *in vivo*, where KI/KI h-Ras lines were capable of enhancing tumor formation kinetics by ~5.5-fold over KI/KI vec cells (Fig4D). Furthermore, analysis of proliferative markers within these tumors found an increase in the number of Ki67-positive cells in the KI/KI h-Ras tumors, demonstrating the enhanced proliferative capacity of these cells (Fig4E). Collectively, these data provide evidence to support the role of cyclin D1b as critical oncogenic ‘hit’, functioning to promote cellular transformation and bypass of senescence programs initiated by the further oncogenic insult.

Passage-matched *Ccnd1*^+/+^ or *Ccnd1*^*KI*^^*/*^^*KI*^ MAF lines were infected with lentivirus containing vector control or h-Ras constructs. Cells were selected with puromycin for 14 days and assayed for β-galactosidase activity. DAPI serves as a control for cell number. Images were taken at 200× magnification with insets taken at 400× magnification.

The indicated cell lines were grown in complete media and assayed for h-Ras expression via immunoblot. Lamin B serves as a loading control.

Cells were plated in soft agar and allowed to grow for a period of 3 weeks. Plates were then washed, fixed, and stained with 0.01% crystal violet. Colonies greater than 50 μm were counted and are plotted (top). Representative images of colony growth are shown for each cell line. Images were taken at 40× with insets taken at 200× magnification.

Indicated MAF lines were injected subcutaneously into the flanks of nude mice as in (A), and tumor incidence is plotted as % of tumor-free mice over time (*n *=* *10).

Indicated tumors were harvested, fixed, and stained for the proliferative marker mKi67. Three random fields from each tumor were counted for mKi67 positivity, and are plotted as a % of total cell number, *n *=* *5 (right: representative images). Images were taken at 400× magnification. Data information: Boxes highlight area of magnified images. Error bars represent ± SEM, and statistical significance was determined using a Student's *t*-test. ***P <* *0.001.

### Genetic switch to cyclin D1b induces serum independence

**Figure 5:**
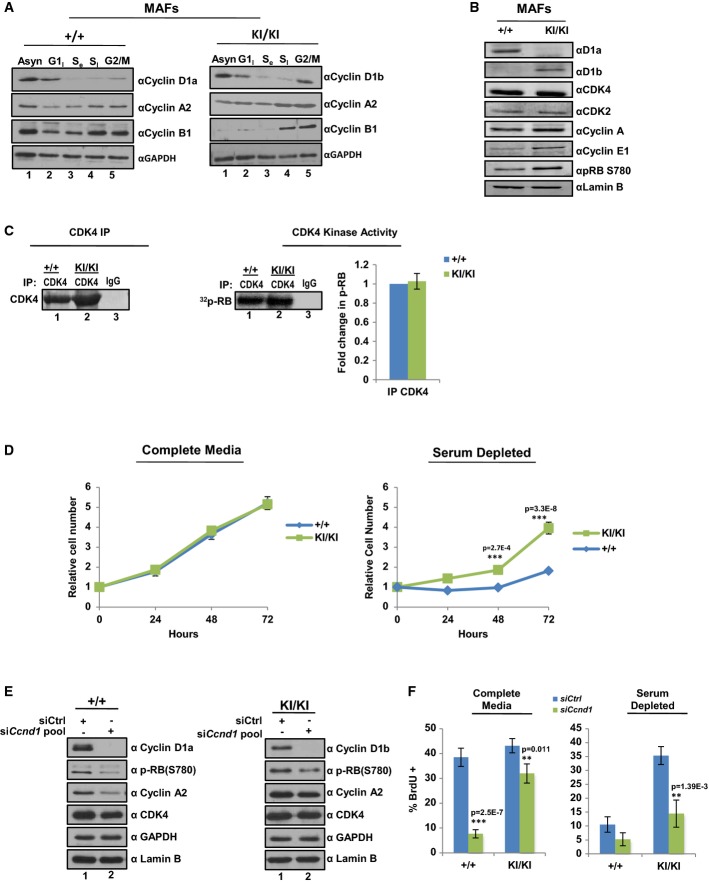
Genetic switch to cyclin D1b promotes serum independence

The concept that endogenously derived cyclin D1b induces transformation is significant and highlights distinctions from cyclin D1a, which requires overexpression to serve as an oncogene. Given the unexpected nature of these findings, *Ccnd1*^*KI/KI*^ cells were used to characterize the molecular consequence of cyclin D1b expression. To examine cyclin D1b distribution/stability in the absence of a constitutively active promoter, *Ccnd1*^+/+^ and *Ccnd1*^*KI/K*I^ cells were arrested in each phase of the cell cycle and expression of individual cyclin D1 isoforms determined. As shown in Fig5A, cyclin D1a levels peaked in G1, and diminished as a function of cell cycle progression (cyclin A2 and cyclin B1 serve as late S and G2/M phase-specific controls). Cyclin D1b expression mimicked that of cyclin D1a, suggesting that both isoforms are regulated in a similar fashion throughout the cell cycle. These data thus demonstrate that the oncogenic activity governed by cyclin D1b is independent of aberrant expression in later phases of the cell cycle.

*Ccnd1*^+/+^ and *Ccnd1*^*KI*^^*/*^^*KI*^MAF lines were arrested in G1, S, and G2/M phases of the cell cycle and expression of cyclin D1 isoforms determined by immunoblot. cyclin A2 and cyclin B1 serve as phase-specific cell cycle controls.

Cells were plated in serum-proficient media and protein lysates generated from each genetic line. Individual D1 cyclins were immunoblotted to verify genetic identity along with other essential cell cycle components. Lamin B serves as a control.

Indicated cells lines were immunoprecipitated for CDK4 and analyzed for their ability to incorporate ^32^P-ATP into full-length RB substrate. Radioactive counts were normalized to CDK4 activity of +/+ cell lines and adjusted for efficiency of CDK4 pull down as determined by densitometry. Data is representative of three independent biological replicates.

Indicated cell lines were plated in serum-proficient (10%) or serum-deficient (1%) media and total cell number counted at 24, 48, and 72 h in biological triplicate.

*Ccnd1*^+/+^ and *Ccnd1*^*KI*^^*/*^^*KI*^ MAF lines were transfected with a validated pool of siRNA directed against the N-terminus of the murine cyclin D1 transcript or control siRNA in full serum. 48 h post-transfection cells were harvested and analyzed for biochemical markers of cell cycle kinetics via immunoblot. GAPDH and Lamin B serve as controls.

Cells were treated as (E), and incubated in the indicated serum concentration for 48 h. Cells were treated with BrdU for 1 h prior to harvesting and then stained for BrdU incorporation. Three random fields from each of three biological triplicates were counted for BrdU incorporation via immunofluorescence and data is represented as percent positive/total cell number. Data information: Error bars represent ± SEM, and statistical significance was determined using a two-tailed Student's *t*-test. **P <* *0.01, ***P <* *0.001.

To further explore the consequence of cyclin D1b, the impact on cell cycle kinetics was determined. As shown in Fig5B, individual MAF lines exclusively expressing cyclin D1a (*Ccnd1*^+/+^) or cyclin D1b (*Ccnd1*^*KI/KI*^) demonstrated comparable expression of G1 and S phase CDK and cyclin components. RB phosphorylation at Ser-780 (an established site of CDK4/6 kinase activity) was maintained in *Ccnd1*^*KI/KI*^ cells, indicating that CDK4/6 kinase activity is preserved in cyclin D1b-expressing cells. This was further validated in tissues derived from age-matched *Ccnd1*^+/+^ and *Ccnd1*^*KI/KI*^ animals (Supplementary Fig S3A), which revealed that phosphorylation of RB at S780 is comparable between +/+ and KI/KI tissues, further suggesting that cells expressing cyclin D1b maintain active CDK4/6 complexes. To more directly address this concept, the ability of cyclin D1 isoforms to interact with and activate CDK4 was determined in both +/+ and KI/KI models. Consistent with previous reports, both cyclin D1a and cyclin D1b effectively interact with CDK4 (Lu *et al*, 2003) (Supplementary Fig S3B). CDK4 kinase assays uncovered a similar ability of CDK4 to phosphorylate RB in the presence of cyclin D1a (+/+ cells), or cyclin D1b (KI/KI cells, Fig5C). These findings are consistent with growth studies that demonstrated nearly identical growth rates in serum-proficient conditions (Fig5D, left). However, when challenged with 1% serum, there was a dramatic difference between the growth potential of *Ccnd1*^+/+^ and *Ccnd1*^*KI/KI*^ cells (Fig5D, right). Parallel studies analyzing active S phase via bivariate flow cytometry validated the growth patterns in both serum-proficient (10%) and serum-deficient (1%) conditions (Supplementary Fig S3C), demonstrating that cyclin D1b expression supports growth in serum-depleted conditions independent of aberrant expression throughout the cell cycle.

To define the dependence of such phenotypes on cyclin D1b expression, a validated pool of siRNA targeting the N-Terminus of murine cyclin D1 (common to both isoforms) was transfected into +/+ and KI/KI MAF lines and assessed for markers of proliferation. As shown in Fig5E, the introduction of siRNA resulted in a dramatic reduction of both cyclin D1a and cyclin D1b. In *Ccnd1*^+/+^ cells, loss of cyclin D1a resulted in a dramatic reduction in the proliferative markers p-RB(S780) and cyclin A and correlated with 80% reduction in bromodeoxyuridine (BrdU) incorporation under these conditions (Fig5F). Interestingly, loss of cyclin D1b in *Ccnd1*^*KI/KI*^ cells resulted in only modest reduction of p-RB(S780) and cyclin A, correlating with only ~20% reduction in BrdU incorporation (Fig5E and F, left). Of importance, cyclin D1b expression was required for growth in serum independent conditions, wherein loss of cyclin D1b resulted in a substantial reduction in BrdU incorporation as compared to control (Fig5F). These findings strikingly reveal an independent capacity of endogenous cyclin D1b to confer serum independence, consistent with capacity of cyclin D1b to drive tumor formation *in vivo*.

### Cyclin D1b expression promotes double-strand breaks and PARP1 activity

**Figure 6:**
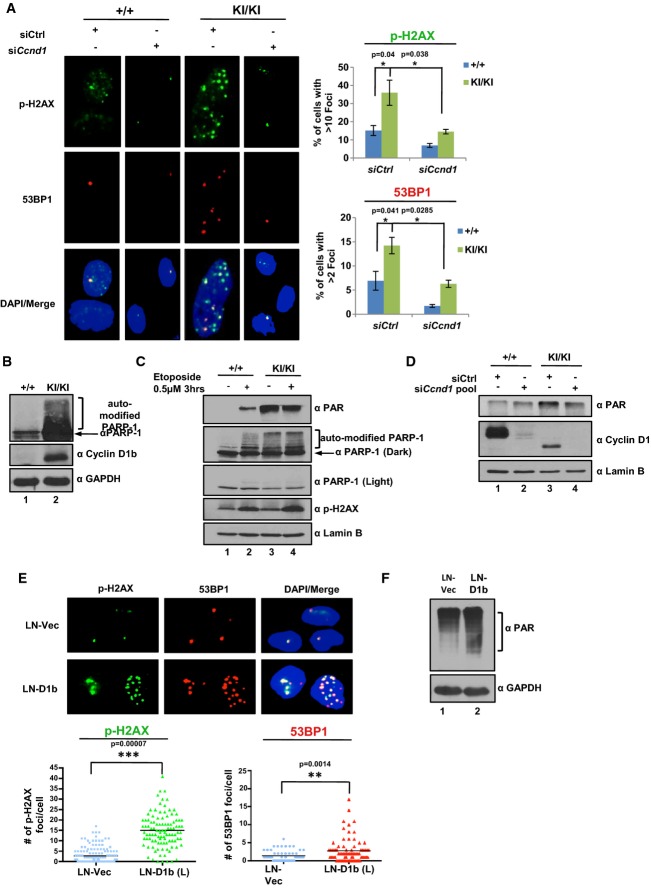
Cyclin D1b expression promotes double-strand breaks and PARP-1 activity

Given the collective observations above that cyclin D1b elicits divergent effects on cellular transformation, and clinical observations that cyclin D1b expression is associated with therapeutic resistance, it was imperative to challenge the impact of therapeutic intervention and or genotoxic stress on cyclin D1b-driven tumor cells. Initially, the presence of DNA damage was assessed in *Ccnd1*^+/+^ and *Ccnd1*^*KI/KI*^ by quantifying intrinsic p-H2AX and 53BP1 foci, established markers of DNA breaks and known metrics for genome integrity. As expected, a majority of the *Ccnd1*^+/+^ cells showed a low frequency of both p-H2AX and 53BP1 foci/cell (Fig6A). In contrast, cells expressing endogenous cyclin D1b harbored increased double-strand breaks, demonstrated by the enhanced prevalence of p-H2AX and 53BP1 foci (Fig6A, quantified right). These data were confirmed in an independently derived *Ccnd1*^*KI/KI*^ cell line (Supplementary Fig S4A), suggesting that *Ccnd1*^*KI/KI*^ cells maintain heightened intrinsic DNA damage. Furthermore, cyclin D1b was required to maintain markers of DNA damage, as loss of cyclin D1b expression reduced the levels of both p-H2AX and 53BP1 foci to those found in *Ccnd1*^+/+^ control cells (Fig6A, quantified right), suggesting that cyclin D1b expression is associated with markers of DNA damage.

*Ccnd1*^+/+^ and *Ccnd1*^*KI*^^*/*^^*KI*^ cells were transfected with a pool of siRNA directed against the N-terminus of the murine cyclin D1 transcript or scramble control for 48 h (as in Figure5). Cells were then fixed and stained for markers of double-strand breaks (p-H2AX and 53BP1, 400× objective). Total number of foci for each cell was quantified and represented as the % of cells with > 10 foci/cell (p-H2AX) or % of cells with > 2 foci/cell (53BP1). Error bars represent ± SEM.

*Ccnd1*^+/+^ and *Ccnd1*^*KI*^^*/*^^*KI*^MAF lines were grown in serum-proficient media for 24 h and probed for expression of auto-modified PARP1 via immunoblot. Gapdh and cyclin D1b serve as controls.

Cells were plated as in (B) and treated with 0.5 μM etoposide for 3 h. Cells were harvested and then probed for markers of DNA damage via immunoblot. Lamin B serves as a control.

Indicated MAF lines were treated as in (A) and harvested 48 h post-transfection. Cells were analyzed for total PAR levels via immunoblot. cyclin D1 levels serve as siRNA validation controls.

cyclin D1b expression was induced in the prostate cancer cell line LNCaP (previously described (Augello *et al*, 2014)) and stained for p-H2AX and 53BP1 foci as in (A) via immunofluorescence (400× objective). Total number of foci/cell is reported for LNCaP vector control and cyclin D1b-expressing isogenic pairs in biological triplicate.

Isogenic pairs from (E) were grown in serum-proficient media for 24 h, harvested, and probed for total PAR levels via immunoblot. Data information: Statistical significance was determined using a two-tailed Student's *t*-test.*P <* *0.05 **P <* *0.01, ***P <* *0.001.

To further explore this concept, levels of auto-modified PARP-1 (known to increase in response to DNA damage) were assayed in *Ccnd1*^+/+^ and *Ccnd1*^*KI/KI*^ lines. As shown in Fig6B, auto-modified PARP-1 was low in *Ccnd1*^+/+^ control cells (lane 1), consistent with the low frequency of p-H2AX foci. Conversely, *Ccnd1*^*KI/KI*^ cells show dramatically heightened levels of PARP-1 activity (lane 2 and Supplementary Fig S4B), further demonstrating that cyclin D1b expression promotes intrinsic DNA damage. To compare intrinsic DNA damage signals to those induced in response to exogenous genomic insult, control and *Ccnd1*^*KI/KI*^ cells were treated with etoposide, known to induce double-strand breaks, and assayed for markers of DNA damage response. Cyclin D1a-expressing cells (*Ccnd1*^+/+^) showed low levels of PARP-1 activity, which were enhanced in the presence of genotoxic stress (Fig6C lanes 1, 2). Increased PARP-1 activity was not due to a total increase in PARP-1, but correlated with enhanced poly-ADP-ribose (PAR) and p-H2AX expression, consistent with initiation of a DNA damage response. In contrast, *Ccnd1*^*KI/KI*^ cells showed high PARP-1 activity in the untreated setting—strikingly, no further increase in PARP-1 activity or PAR levels was observed after etoposide treatment (Fig6C lanes 3 and 4). Consistent with the loss of DNA damage markers after cyclin D1b depletion, loss of cyclin D1b resulted in a decrease in total PAR levels (Fig6D), which was not observed with cyclin D1a loss in *Ccnd1*^+/+^ cells. These data indicate that PARP-1 is activated in cells that shift from cyclin D1a to cyclin D1b, thus further suggesting that cyclin D1b destabilizes genome integrity.

To ensure that the observed intrinsic DNA damage signals seen in *Ccnd1*^KI/KI^ cells were not due to a defect in p53 signaling, the p53 target gene *Cdkn1a* (p21) was analyzed before and after etoposide treatment. As shown in Supplementary Fig S5A, an increase in *Cdkn1a* transcript is readily seen as early as three hours post-treatment in both *Ccnd1*^+/+^ and *Ccnd1*^*KI/KI*^ lines, which correlated with an increase of p21 protein (Supplementary Fig S5B), suggesting that *Ccnd1*^*KI/KI*^ cells maintain a functional p53 pathway. As a further control for specificity and relevance to human disease, the ability of cyclin D1b to promote intrinsic DNA damage signals was determined in human prostate cancer models (a tumor type known to shift from cyclin D1a to cyclin D1b (Comstock *et al*, 2009)). Utilizing previously described isogenic models with or without cyclin D1b expression (Augello *et al*, 2014), it was observed that cells expressing cyclin D1b harbored a greater number of p-H2AX and 53BP1 foci/cell than control (Fig6D, right). The induction of cyclin D1a alone had no significant effect on the prevalence of either p-H2AX or 53BP1 foci (Supplementary Fig S6). Furthermore, PARP-1 activity was heightened in cyclin D1b-expressing cells, mirroring the intrinsic DNA damage signals seen in *Ccnd1*^*KI/KI*^ cell lines. Collectively, these data highlight novel actions of cyclin D1b in fostering intrinsic DNA damage signals in multiple model systems, and offer novel insight into the oncogenic action of this cyclin D1 isoform as it relates to human disease.

### Cyclin D1b expression sensitizes to senescence induced by combined therapeutic challenge

**Figure 7:**
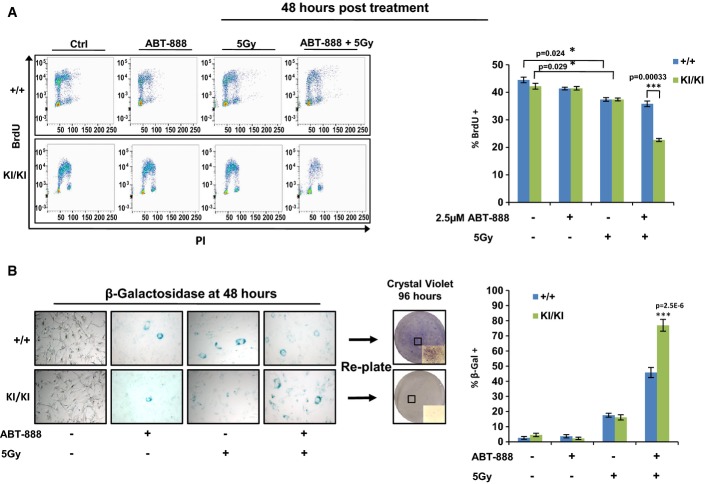
Cyclin D1b expression sensitizes cells to senescence induced by therapeutic challenge

The switch from cyclin D1a to cyclin D1b is observed in numerous human malignancies and is associated with poor prognosis. Despite this knowledge, delineation of mechanisms to target cyclin D1b-positive/cyclin D1b-driven tumor cells remains elusive. Building on the observations herein, it was hypothesized that therapeutically targeting DNA damage and PARP pathways individually or in combination would sensitize cells to cell cycle arrest and/or cell death. Thus, control (*Ccnd1*^+/+^) and *Ccnd1*^*KI/KI*^ cells were treated either with the PARP inhibitor ABT-888, 5 Gy of radiation, or a combination and analyzed for cell cycle progression. As shown in Fig7A, both *Ccnd1*^+/+^ and *Ccnd1*^*KI/KI*^ cells showed similar proliferative kinetics when unchallenged in normal growth conditions, and neither cell type was affected by ABT-888 alone. In *Ccnd1*^+/+^ cells, treatment with 5 Gy IR diminished BrdU incorporation by ~10%, which was unchanged with pre-treatment of ABT-888. Conversely, while *Ccnd1*^*KI/KI*^ cells responded similarly to 5 Gy IR alone, combination of IR and ABT-888 dramatically inhibited BrdU incorporation (Fig7A, quantified right). The reduction in proliferative capacity was associated with altered morphology, indicative of senescence. As such, cells were treated as above and then assayed for senescence-associated β-galactosidase (β-Gal) activity. As shown, only a small fraction of cells stained positive in the control and ABT-888-treated conditions (Fig7B, quantified bottom), and IR alone increased the presence of β-Gal activity equally in both cell models. However, there was a pronounced induction of β-Gal staining after combination treatment in the *Ccnd1*^*KI/KI*^ line, nearly double that of the *Ccnd1*^+/+^ control. Re-plating both the *Ccnd1*^+/+^ and *Ccnd1*^*KI/KI*^ cells (which were co-treated) determined that while control cells were able to reengage the cell cycle and repopulate the dish after 96 h, *Ccnd1*^*KI/KI*^ cells were markedly unable to do so. These data thus demonstrate that the intrinsic DNA damage and heightened PARP-1 activity seen in cyclin D1b-expressing cells may be exploited to thwart growth of this cell type via combined therapeutic challenge.

*Ccnd1*^+/+^ and *Ccnd1*^*KI*^^*/*^^*KI*^MAF lines were grown in serum-proficient media for 24 h. Cells were then treated with control DMSO or 2.5 μM of the PARP inhibitor ABT-888. One hour post-treatment, the indicated lines were treated with 5 Gy of radiation. Cells were then allowed to recover for 48 h, after which BrdU was added for 1 h, and then harvested for bivariate flow cytometry. Representative traces for each condition are shown (left) and BrdU incorporation of biological triplicates was quantified (right).

Cells were plated and treated as in (A). Forty-eight hours post-treatment, cells were fixed and stained for markers of senescence (β-galactosidase activity). Cells positive for the staining (blue) were quantified for each condition (400× magnification) and reported as a percentage of the total population (right). Plates treated in parallel with both IR and ABT-888 were harvested at 48 h via 1× trypsin and re-plated in serum-proficient media. Cells were allowed to grow for 96 h and were then stained with crystal violet (4× objective, with inset at 200× magnification, boxes highlight area of magnified images). Data information: Error bars represent ± SEM, and statistical significance was determined using ANOVA (A) or a two-tailed Student's *t*-test (B). *P <* *0.05, ***P <* *0.001.

## Discussion

**Figure 8:**
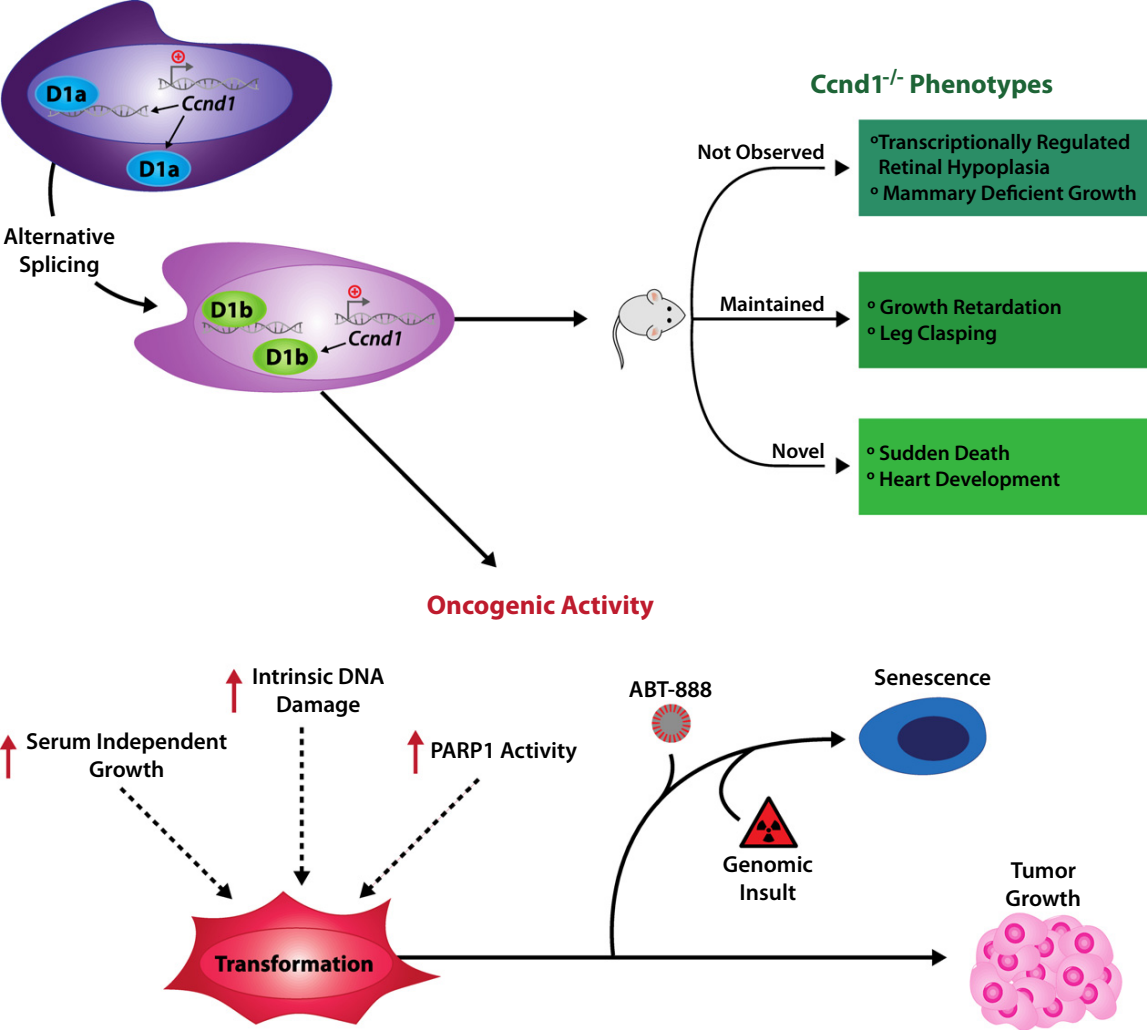
Model of cyclin D1b function in development and tumorigenesis Top: Comparison of the observed phenotypes noted in the *Ccnd1*^*KI/KI*^ and *Ccnd1*^*−/−*^ mice (Sicinski *et al*, 1995). Bottom: Summation of the pro-oncogenic phenotypes associated with cyclin D1b expression in cell-based models.

Here, a novel genetically engineered mouse model was used to convert cyclin D1a to cyclin D1b, allowing for first-in-field analysis of cyclin D1b *in vivo* and under control of the endogenous promoter (Fig1). Notably, cyclin D1b expression phenocopied only a selected subset of *Ccnd1*^−/−^ mouse aberrations (Fig2), supporting the concept that cyclin D1b harbors distinct functions from that of cyclin D1a. Parallel studies utilizing isoform-specific models revealed the oncogenic potential of cyclin D1b, which further cooperated with known oncogenes to promote cellular transformation and tumor growth at high frequency (Figs3, 4 and 5), was associated with persistent DNA damage signals (Fig6). Furthermore, *Ccnd1^KI/KI^* cells were sensitized to cell cycle arrest and senescence though combined therapeutic intervention (Fig7), providing the first preclinical evidence of mechanisms to target cyclin D1b-expressing tumors. Collectively, this new model system unveiled novel functions of cyclin D1b in both development and tumorigenesis (Fig8) and identified means to target tumor cells that depend upon cyclin D1b expression.

Genetic switching from cyclin D1a to cyclin D1b unmasked unique functions of this isoform in development and homeostasis. Data herein demonstrated that *Ccnd1*^*KI/KI*^ mice displayed several notable phenotypes consistent with genomic loss of cyclin D1 which include (1) delayed post-natal growth kinetics, (2) neuro/muscular limb dysfunction, and (3) low frequency of early onset death. Interestingly, *Ccnd1*^*KI/KI*^ animals displayed no evidence of the hallmark retinal hypoplasia and mammary deficient growth phenotypes seen in multiple models of *Ccnd1* loss (Sicinski & Weinberg, 1997; Landis *et al*, 2006). Collectively, these data demonstrate that cyclin D1b harbors overlapping but non-redundant functions with that of cyclin D1a, and provide the first *in vivo* evidence of divergent biochemical action of this variant. Insight into possible underlying mechanisms of cyclin D1b function was gained from previous models of cyclin D1 deregulation aiming to dissect the cell cycle from transcriptional functions of cyclin D1a. In an identical genetic background, knock-in of the kinase activation dead mutation (K112E) of cyclin D1 (*Ccnd1*^*KE/KE*^) rescued the retinal hypoplasia and mammary phenotypes of the *Ccnd1*^−/−^ mouse, but not the growth retardation, or leg clasping phenotypes (Landis *et al*, 2006). These data are consistent with the known reliance on the transcriptional functions of cyclin D1 for retinal and mammary development and demonstrate that the cell cycle functions of cyclin D1 are required to rescue other phenotypes seen upon *Ccnd1* loss. The similarity between the *Ccnd1*^*KE/KE*^ and *Ccnd1*^*KI/KI*^ models thus suggests that endogenous production of cyclin D1b is not sufficient to fully engage the cell cycle machinery (CDK4/6 complexes) in normal tissue, and supports the concept that cyclin D1b functions as a potent transcriptional regulator *in vivo*. Furthermore, the novel phenotypes of *Ccnd1*^*KI/KI*^ mice (in echocardiographic ventricular wall thickness/mass and sudden death) indicate that the transcriptional functions of cyclin D1b are divergent from that of full-length cyclin D1a. Given the established transcriptional roles of cyclin D1b in disease progression, future efforts will be aimed at defining the underlying mechanisms behind cyclin D1b-mediated transcriptional regulation and its role in both normal and malignant cellular processes.

In addition to the unique developmental functions of cyclin D1b, novel observations were made regarding the impact of cyclin D1b on neoplastic transformation. Data herein demonstrated that cyclin D1b induced transformation of immortalized cells generate tumors with high frequency (Fig4). In contrast, wild-type cells were non-malignant, and forced overexpression of cyclin D1a (+/+ cells) or cyclin D1b (KI/KI) cells had little impact on the tumorigenicity of either line, demonstrating that the a major oncogenic function of cyclin D1 is likely manifest through the induction of cyclin D1b rather than its overexpression. Furthermore, only cells expressing cyclin D1b were able to efficiently proliferate after the induction of h-Ras, this is, in stark contrast to those expressing cyclin D1a which rapidly underwent oncogene-induced senescence (as determined via β-galactosidase activity). These data suggest that the induction of cyclin D1b can act as a critical ‘hit’ during tumorigenesis which can facilitate the accumulation of further oncogenic events which cooperate to drive tumor formation and progression. Collectively, these data thus provide the first genetic evidence for cyclin D1b as an oncogene, and implicate cyclin D1b as a potential biomarker of disease progression.

Although hyperplastic and neoplastic lesions were observed in some *Ccnd1*^*KI/KI*^ mice, the frequency of occurrence did not differ from that of *Ccnd1*^+/+^ animals. While further studies are needed, the observed frequency of neoplasms is consistent with previous models of cyclin D1a deregulation in similar backgrounds (Wang *et al*, 1994). It is notable that even transgenic models of forced cyclin D1a overexpression show quite limited capacity for tumor formation, which is latent, and requires co-expression of the Erbb2 oncogene for robust tumor induction in younger mice (Wang *et al*, 1994). Furthermore, transgenic murine models harboring forced expression of mutations in the cyclin D1a PEST domain (known to promote cyclin D1a stability) require greater than 12 months for significant tumor induction (Gladden *et al*, 2006). Collectively, these data suggest that cellular transformation that results due to deregulation of cyclin D1a is a latent event. Given the potent functions of cyclin D1b on tumor formation described above, it will be essential to define the frequency and distribution of cyclin D1b induction across multiple tumor types, to more completely understand the contribution of this isoform in malignant transformation and tumor progression.

The finding that endogenous expression of cyclin D1b independently promotes transformation of immortalized cells represents a significant advance in our understanding of the differing oncogenic capacity of cyclin D1 variants in tumor biology. Further, an unexpected link between cyclin D1b and DNA damage signals was discovered, wherein cyclin D1b-expressing cells harbor elevated levels of p-H2AX and 53BP1 foci as well as PARP-1 activity (Fig1). Previous *in vitro* models of cyclin D1 depletion described a critical role of cyclin D1a in the DNA damage response pathway, wherein cyclin D1a was necessary for BRCA1 recruitment to sites of damage, and initiation of repair cascades (Jirawatnotai *et al*, 2011). Furthermore, mutations in the cyclin D1 PEST domain have been shown to promote cyclin D1a stability, which resulted in inappropriate expression throughout the cell cycle, culminating in aberrant assembly of replication machinery and DNA damage signals (Aggarwal *et al*, 2007). While it is tempting to speculate that damage signals observed in cyclin D1b-expressing cells originated from either of these mechanisms, data herein suggest that the pathways are disparate. Indeed, analyses of cyclin D1b expression across the cell cycle closely mimicked that of cyclin D1a, demonstrating that despite lacking PEST encoding sequences, cyclin D1b is regulated in a similar temporal and kinetic manner to that of cyclin D1a (Fig5). Additionally, no difference was found in the proliferative indices between cyclin D1b-expressing and wild-type cells post-ionizing radiation (Fig7), suggesting that DNA damage repair pathways (responsible for resolution of double-strand breaks) are not compromised in cyclin D1b-expressing cells. These data thus support the concept that the mechanisms underlying the intrinsic damage signals in cyclin D1b-expressing lines are likely independent of major defects in DNA damage or cell cycle pathways. Interestingly, recent evidence suggested that the functions of PARP-1 extend beyond DNA damage repair (Luo & Kraus, 2012). Critical roles in transcriptional regulation have recently been attributed to PARP-1 activation, which impact a diverse array of biological processes (including inflammation, cell growth, survival, and apoptosis) (Luo & Kraus, 2012; Schiewer *et al*, 2012a,b; Koh *et al*, 2005; Rajamohan *et al*, 2009). It has been proposed that PARP-1 functions as a cellular ‘rheostat’, wherein the intensity of cellular stress dictates the level of PARP-1 activation and subsequent biological response (Luo & Kraus, 2012). Thus, given the tumor-associated transcriptional functions of cyclin D1b, and the herein established link between PARP-1 activity and cyclin D1b expression, it will be critical to determine whether the two transcriptional networks cooperate to promote transformation. Future efforts will focus on the defining the dependence of cyclin D1b-expressing cells on PARP-1 transcriptional functions, and delineate the networks responsible for both heightened PARP-1 activity and p-H2AX foci.

Recent evidence has demonstrated that PARP-1 activity is enhanced in selected tumors and likely contributes to tumor progression (Brenner *et al*, 2011; Do & Chen, 2013; Luo & Kraus, 2012; Schiewer *et al*, 2012a,b; Kedar *et al*, 2012; Horton & Wilson, 2013; Horton *et al*, 2014; Schiewer & Knudsen, 2014). This concept is supported by clinical evidence demonstrating PARP-1 suppression can delay tumor progression (Fong *et al*, 2010). Consistent with these findings, cyclin D1b-expressing cells were sensitized to cell cycle arrest and senescence by combined PARP inhibition and ionizing radiation. The robust response observed with combination treatment in cyclin D1b-expressing lines indicates that PARP function is required for initiation of an efficient repair response to genotoxic stress in these cells. Given the heightened p-H2AX foci level and sustained PARP-1 activation state observed in cyclin D1b-expressing lines, these cells appear reliant on PARP-1 signaling cascades for genomic maintenance. Consonantly, a similar dependence on PARP-1 activity has been demonstrated clinically, where PARP-1 inhibition effectively limited tumor growth in *BRCA2/1*-mutated tumors (Fong *et al*, 2010), and showed single-agent efficacy in patients with advanced castration-resistant prostate cancer (a tumor type and stage known to express high levels of cyclin D1b) (Fong *et al*, 2010; Sandhu *et al*, 2010). Taken together, cyclin D1b could potentially serve as a novel biomarker of response to therapeutic intervention, and critical next steps will aim to characterize key interactors and downstream pathways in cyclin D1b tumors, which govern this response.

In summary, the present study describes the first-in-field model of the switch from cyclin D1a to cyclin D1b and demonstrates overlapping but non-redundant functions of this isoform *in vivo*. Further, these data provide the first genetic evidence for cyclin D1b as an oncogene and uncovered novel links between cyclin D1b expression and the DNA damage and PARP1 networks. These unexpected results provided the first preclinical evidence of a method to specifically target cyclin D1b-expressing tumors and serve as the rationale to develop cyclin D1b expression as a novel biomarker of therapeutic response.

## Materials and Methods

### Generation of *Ccnd1*^*KI/KI*^ mice

Design of the knock-in targeting constructs that humanize the murine *Ccnd1* locus to produce cyclin D1b in the C57BL6/129 mixed background is described in Fig1A. Generation and cloning of targeting constructs, embryonic injection and selection, clone verification, and generation of chimeric mice were conducted by InGenious Laboratories LLC. Validation of the cyclin D1b knock-in allele was performed using PCR from genomic DNA (100 ng) using the primers (Murine *Ccnd1* exon 3 – 5′AGT GCC ACT TAG GTG TCT CCA3′, Murine *Ccnd1* exon 5 – 5′ ACC AGC CTC TTC CTC CAC TT3′, and human *CCND1* intron 4 – 5′ TCT GGA GAG GAA GCG TGT GAG G 3′) (Fig1). Mice were housed in animal facilities within the Kimmel Cancer Center at Thomas Jefferson University, and all protocols utilized for this study were approved by the Institutional Animal Care and Use Committee (IACUC) at Thomas Jefferson University. If not otherwise stated all mice utilized for the study were male, between the ages of 4 and 6 months.

### Immunoblotting

Lysates from either animal tissue (100 μg) or cell lines (20–30 μg) were run on a 10% acrylamide SDSPAGE gel via gel electrophoresis, transferred to a PDVF membrane at 85 V for 1 h in transfer buffer (20% methanol, 0.1% SDS, 0.58% Tris base, 2.4% glycine), blocked in 5% milk buffer (TBST) for 1 h at room temperature, and then were probed with the given antibody overnight at 4°C.

### Antibodies

Antibodies used for immunoblotting, immunohistochemistry, immunofluorescence, and flow cytometry include the following: cyclin D1b (previously described (Comstock *et al*, 2009)), cyclin D1a (Ab-4; NeoMarkers RB-212-P), cyclin A (C-19 sc-596; Santa Cruz), CDK4 (IP C-22 sc-260, IP and immunoblot H-22 sc-601; Santa Cruz), GAPDH (FL-335 sc-25778), pp-RB S780 (C84F6 #3590S; Cell Signaling), PARP (#9542S; Cell Signaling), CDK2 (M2 sc-163; Santa Cruz), cyclin B1 (H-433 sc-752; Santa Cruz), cyclin E1 (sc-198), mKi67 (TEC-3 M7249 DAKO), BrdU (FITC-556028; BD Pharmingen), Lamin B (M-20 sc-6217; Santa Cruz), p21 (C-19 sc-397; Santa Cruz), PAR (4335-amc-050; Trevigen), p-H2AX S129 (IF - #05-636 (Millipore), and WB -20E3 #9718S (Cell Signaling)), p53 (Pab 240 ab26; Abcam), 53BP1 (Novus Biologicals, NB 100-304), h-RAS (C-20 sc-520; Santa Cruz), and cyclin D1 (IP 1:50 immunoblot 1:1,000 04-221; Millipore).

### siRNA and transfection

A validated pool of 4 siRNA constructs directed against the N-terminus of the murine cyclin D1 transcript or scramble control was purchased from Dharmacon (L-042441-00-0020 and D-001810-10-20) and transfected into *Ccnd1*^+/+^ or *Ccnd1*^*KI/KI*^ lines using the Dharmacon 1 transfection reagent. Forty-eight hours post-transfection, cells were harvested and utilized for immunoblot, growth, and immunofluorescence assays.

### Cell growth curves

Indicated cell lines were plated in 10-cm plates in the conditions indicated above (in biological triplicate). At the indicated time points, cells were harvested via trypsin, washed with 1× PBS, and resuspended in 1 ml of 1× PBS on ice. All labels were then obscured, and live cell number was determined using trypan blue exclusion and a hemocytometer. Blinded cell counts were then averaged and are plotted as an average of three biological replicates. Significance was determined using a Student's *t*-test.

### Flow cytometry

MAF cells were challenged as described above, and adherent and non-adherent cells were collected and fixed with 100% ice-cold ethanol overnight. Proliferative indices were analyzed with bivariate flow cytometry using a 1-hour pulse label of BrdU (Amersham; GE Healthcare Life Science RPN201) before harvest, and propidium iodide staining for cell cycle position (previously described (Schiewer *et al*, 2010)). A BD LSR II flow cytometer was used to sort 10,000 single cells per biological replicate, and FlowJo software was utilized to gate for BrdU incorporation.

### Assessment of mouse *in vivo* cardiac function via echocardiography

The non-invasive technique of transthoracic echocardiography was conducted using a VisualSonics Vevo 770 high-resolution imaging system (Gao *et al*, 2010). Briefly, mice were anesthetized with 1.5% of isoflurane and two-dimensional echocardiographic views of the mid-ventricular short axis obtained at the level of the papillary muscle tips below the mitral valve. A minimum of 400 beats were recorded during each study and analyzed by an independent blinded observer. M-mode measurements of LV internal dimensions (LVID) are determined at the plane bisecting the papillary muscles according to the American Society of Echocardiography leading edge method on 5 heart beats chosen at random by each observer. Values of ejection fraction and fractional shortening are obtained automatically (Gao *et al*, 2010).

### Generation of primary cell lines and cell culture

Age-matched mice (3–4 months old) were sacrificed, washed in 70% EtOH and peritoneum harvested. Under sterile conditions, tissue was washed 2× in 1× PBS and diced using a sterile blade. Tissue was dissociated using collagenase (2 mg/ml in L-15 media with DNAse) for 45 min at 37°C with gentle shaking. Tissue was then pelleted, washed with warm 1× PBS, and further digested with 1× trypsin (0.25%) for 15 min at 37°C. The trypsin was then neutralized with DMEM containing 10% fetal bovine serum and plated in tissue culture grade plates. One week post-plating, tissue was removed from the plates, and adherent cells were harvested and re-plated. Individual cell lines were then passaged according to a 3T3 protocol.

### Xenografts

Indicated MAF cell lines were cultured as described above, then harvested via trypsin and combined with Matrigel (BD Biosciences, 354234) at a ratio of 1:1. A total of 1 million cells were injected subcutaneously into the flanks of nude mice (*n* = 10/genotype), and tumor growth/development was measured twice weekly over a period of 1 year. Tumor growth was monitored with electronic calipers weekly, and mice were sacrificed when tumor volume reached 750 mm^3^ or 3 weeks post-tumor detection. Individual tumors were removed from mice and immediately fixed in 10% buffered formalin overnight. Tumor sections were then processed and embedded into paraffin blocks and cut into individual 5-μM sections. Tumor sections were then stained with H&E or immunostained with antisera for the proliferative marker mKi67 as previously described (Augello *et al*, 2014; Comstock *et al*, 2011, 2013). All xenograft studies were performed in accordance with NIH and IACCUC guidelines and were approved by Thomas Jefferson University.

### Cell cycle arrest

MAF lines indicated were plated at ~60% confluence in DMEM containing 10% fetal bovine serum (FBS) and then treated with vehicle (DMSO 0.1%), 5 μg/ml roscovitine (G1 arrest), 1 mM hydroxyurea (early S phase), and 2 μg/ml aphidicolin (late S phase) for 24 h. Cells arrested in G2/M were first treated with 2 μg/ml aphidicolin for 16 h; then, cells were washed with 1× sterile PBS three times, media refreshed, and treated with 50 ng/ml of nocodazole for 8 h. Cells were then harvested via trypsin and lysed using RIPA buffer. Experiments were conducted in biological triplicate.

### p-H2AX and 53BP1 foci counts

Indicated MAF lines were plated on culture grade coverslips and treated as described above. At the indicated time points, cells were washed with 1× PBS, fixed with 3.7% formaldehyde for 10 min at room temp, and stained for p-H2AX foci as previously described (Goodwin *et al*, 2013). Foci were imaged on a Zeiss Confocal Laser Scanning Microscope and counted for at least 50 cells per treatment condition and cell line.

### Histology

Sacrifice of age-matched animals (*n* = at least 4 per genotype) was conducted according to NIH guidelines and in accordance with IACUC-approved protocols at Thomas Jefferson University. Individual organs were harvested and weighed, fixed in 10% neutral buffered formalin, processed, and paraffin-embedded, and 5-μM sections were stained with hematoxylin and eosin. Histopathology and neoplastic features were determined by a board-certified veterinary pathologist.

### CDK4 immunoprecipitation and kinase assay

CDK4 immunoprecipitation and kinase assays were preformed as previously described (Knudsen *et al*, 1998). Immunoprecipitated CDK4 and purified human retinoblastoma protein (1.5 μg per reaction; QED Bioscience #3108) were reconstituted in 40 μl of kinase buffer (Knudsen *et al*, 1998) on ice. The reaction was initiated by the addition of ATP mix (50 μM final, 5 μCi [γ-^32^P] ATP per reaction; Perkin Elmer) and incubated for 30 min at 30°C. Reactions were stopped by the addition of SDS sample buffer, and samples were rocked for 20 min at room temperature and cleared by centrifugation at 15,000 × *g* for 1 min. A total of 20 μl of the total reaction was processed by SDSPAGE on a 10% polyacrylamide gel, and gels were then fixed, stained with Coomassie blue, and dried. Rb phosphorylation was visualized by autoradiography and quantified by Cerenkov counting of the excised band. Total counts per reaction were determined by counting an aliquot of the radiolabeled ATP mix to enable calculation of phosphate incorporation into Rb. Phosphate incorporation was normalized to the immunoprecipitation efficiency of CDK4, as determined by Western blotting and densitometry, to determine the relative kinase activity.

### Soft agar

A total of 5,000 cells suspended in DMEM supplemented with 20% FBS were mixed with 0.70% Bacto agar and plated on a feeder layer of 1.2% agar. After agar was solidified, 3 ml of complete DMEM was added to each 6-cm plate. After a period of 3 weeks, cells were fixed with 3.7% formaldehyde for 10 min at room temperature and stained with 0.01% crystal violet, and colonies greater than 50 μm in diameter were counted. Images were taken at 400× and 200× magnification.

### β-Galactosidase staining and counts

Individual MAF lines were plated on tissue culture grade coverslips and treated as described above. At the indicated time points, cells were fixed with 3.7% formaldehyde and stained for B-galactosidase activity using a Senescence beta-Galactosidase Staining Kit (Cell Signaling #9860S). Cells were stained at 37°C overnight and then counterstained with the nuclear stain DAPI (Invitrogen D3571). Blue (positive) cells were counted in three random fields for each biological replicate, along with total cell number (indicate by DAPI nuclear staining). Data were graphed as a percent positive of total cell population and significance determined using a two-tailed Student's *t*-test.

### Gene expression

Individual cell lines were plated in 10% FBS in DMEM and treated as described above for 3 hours. Cells were then washed and RNA harvested using the TRIzol method (Life Technologies 1196-018). cDNA was generated using 1 ng of purified RNA and the SuperScript VILO enzyme system (Life Technologies 11754-050). Expression of *Cdkn1a* levels was determined using methods previously described (Comstock *et al*, 2011, 2013). Primers used were as follows: *Gapdh –* 5′ AAC TTT GGC ATT GGC AAT GTG GAA GG-3′, 5′ACA CAT TGG GGG TAG GAA CA-3′; and *Cdkn1a* – 5′- GCT GTC TTG CAC TCT GGG GT-3′, 5′-CGT GGG CAC TTC AGG GTT TT-3′.

## Supporting Information

Supplementary Figures S1–S6

Review Process File
