# Supplementary material for: Consequence of the tumor-associated conversion to cyclin D1b
Source: EMBO Mol Med. 2015 Mar 18;7(5):628–47. doi: 10.15252/emmm.201404242 (PMC4492821; doi:10.15252/emmm.201404242)
Supplement: Supplementary file 1 [file emmm0007-0628-sd1.pdf]

Supplemental Table of Contents:

Supplemental Figures………………………………………………………… 2-7

S1: Cyclin D1b knockin mice exhibit normal mammary tissue development postpartum and exhibit low frequency of early onset death…………………………………………………………... 2

S2: Estimated left ventricular mass and diameter is altered in *Ccnd1*$^{KI/KI}$ animals……………………………………………………… 3

S3: Cyclin D1b expressing cells maintain +/+ levels of p-RB *in vivo* and cell cycle entry during serum deprivation……………………....... 4

S4: Validation of intrinsic markers of DNA damage and PARP activity in an independently derived KI/KI MAF line………………… 5

S5: *Ccnd1*$^{+/+}$ and *Ccnd1*$^{KI/KI}$ MAF's are p53 positive and induce the p53 target gene *Cdkn1a* in response to genomic stress……….….. 6

S6. Induction of Cyclin D1a is not associated with markers of DNA damage...7

Supplemental Figure legends S1-S6……………………………………….8-10

Figure S1. Knudsen

A

Phenotype	+/+	+/KI	KI/KI
Ratio of mice which died within 3 months of birth	0/59 (0%)	1/163 (0.6%)	18/55 (32%)

B

Phenotype	+/KI	KI/KI
# of successful pregnancies	28/30	4/6
% of pups surviving to 1 week with birth mother	97.115	0
% of pups surviving to 1 week with foster mother	-	100

C

Ccnd$^{+/+}$ nulliparous — *Ccnd1* $^{+/+}$ post partum — *Ccnd1*$^{KI/KI}$ 3 weeks pregnant

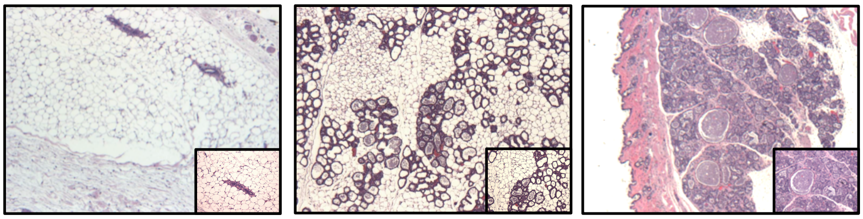

D

Ccnd1$^{-/-}$ Retina

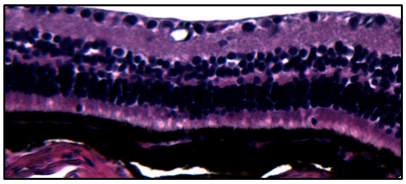

Figure S2. Knudsen

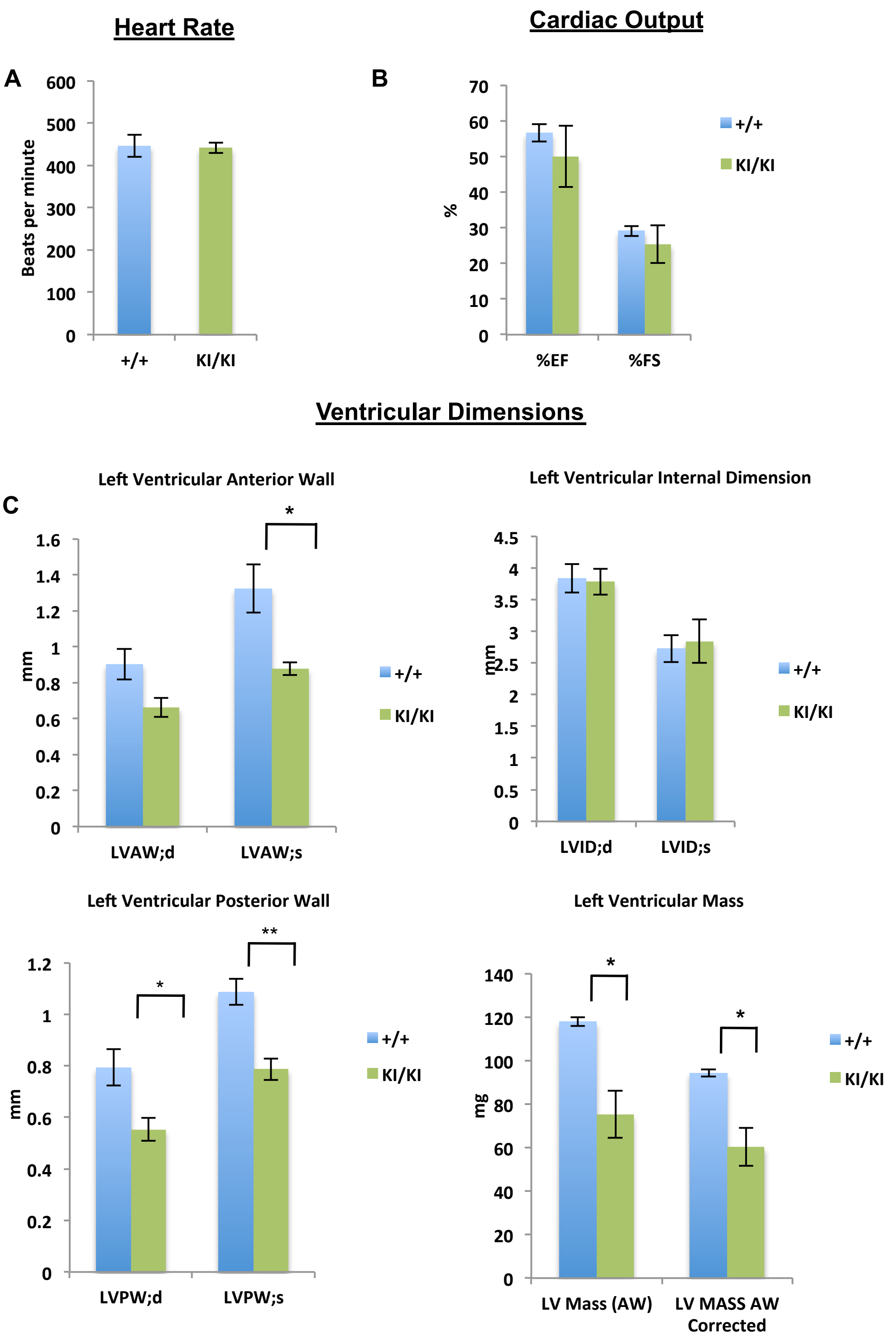

Figure S3. Knudsen

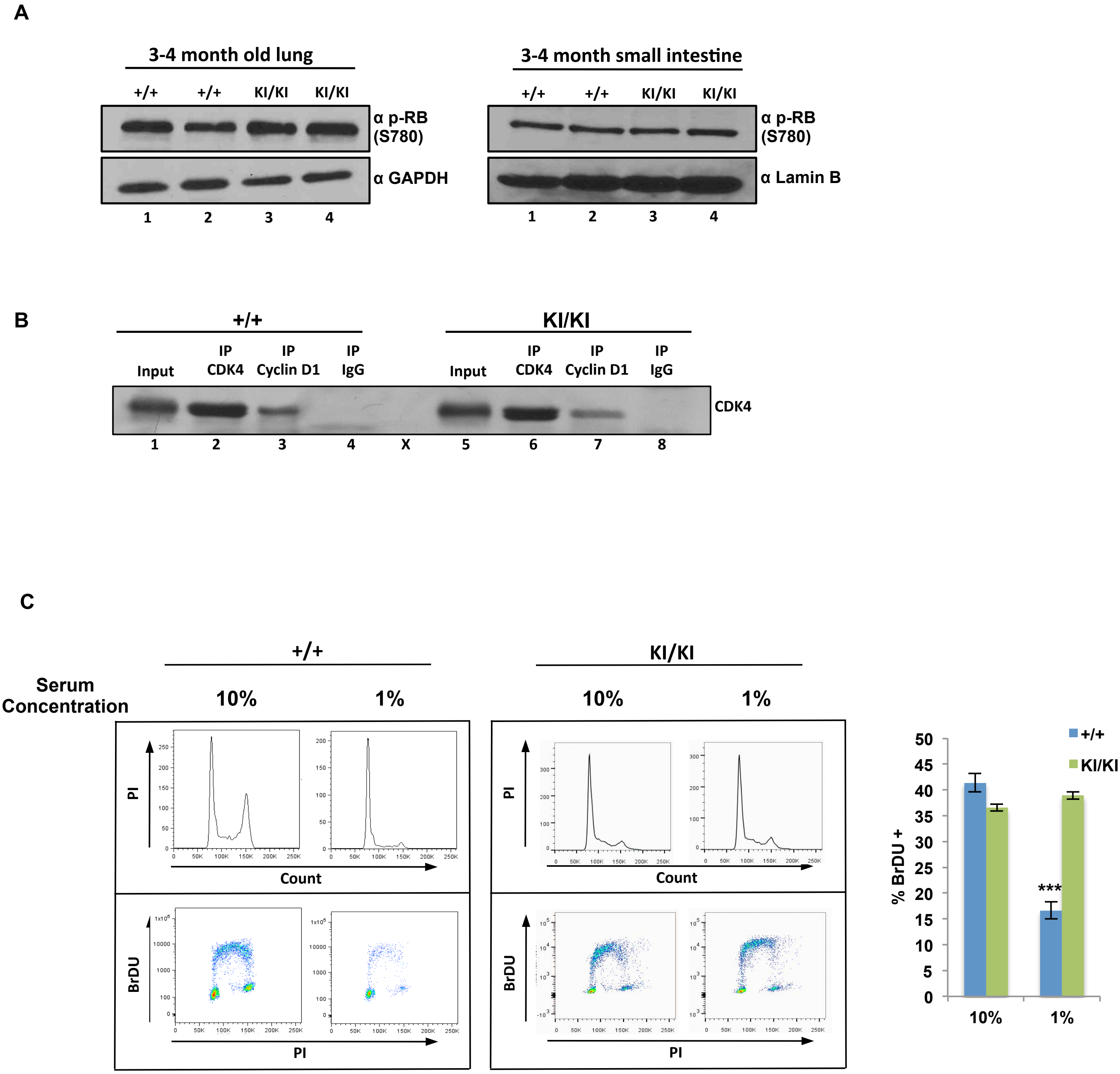

Figure S4. Knudsen

A

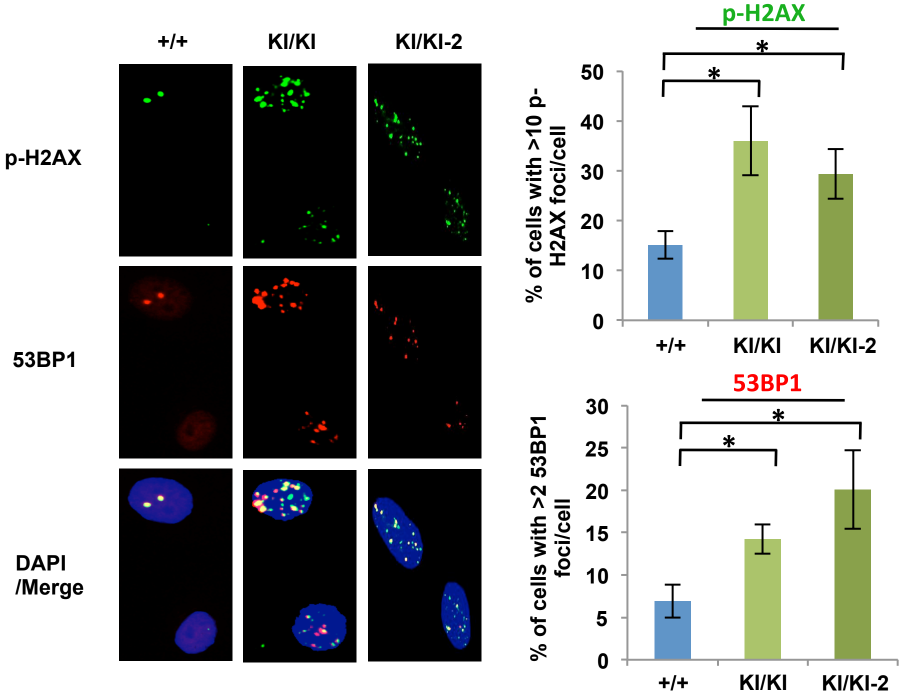

B

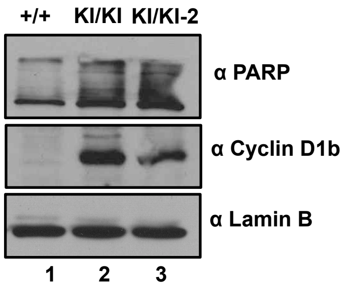

Figure S5. Knudsen

A

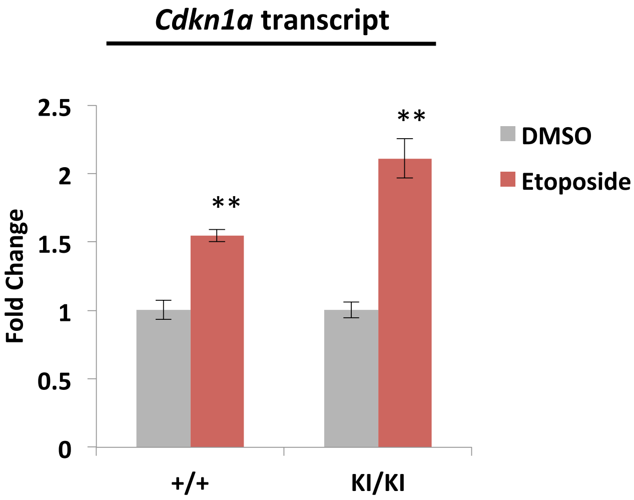

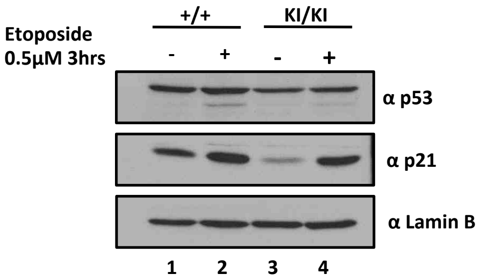

Figure S6. Knudsen

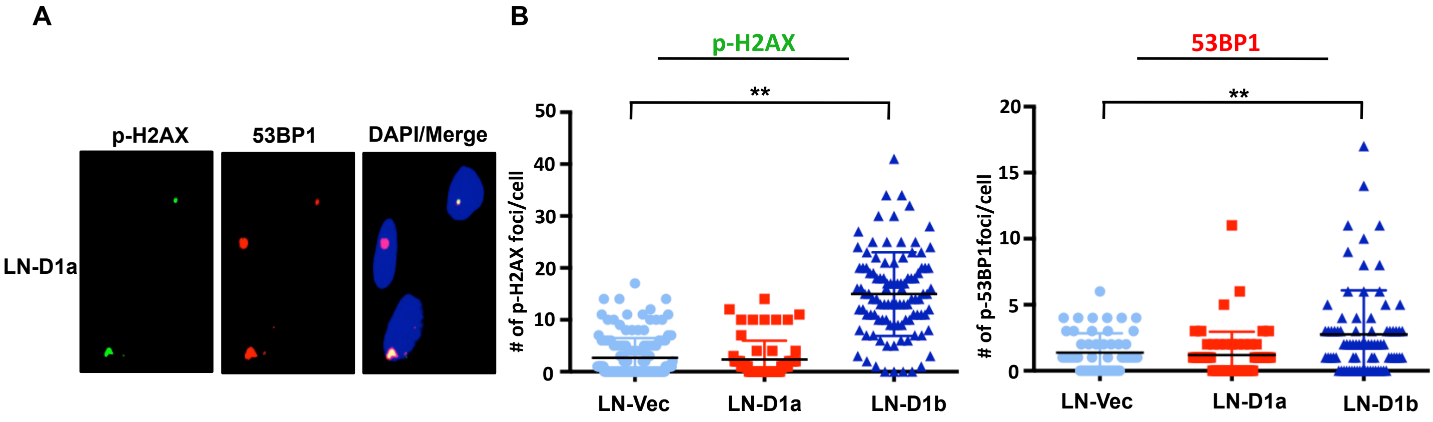

Figure S1. **Cyclin D1b knockin mice exhibit normal mammary tissue development postpartum and exhibit low frequency of early onset death.** A. Summary of breeding characteristics of mice *Ccnd1*$^{+/KI}$ and *Ccnd1*$^{KI/KI}$ mice. B. Quantification of mice that displayed early and sudden onset death before 3 months of age. C. Immunohistochemical analysis of mammary glad development 3 weeks post pregnancy or 1 day post partum. Mammary gland tissue was harvested and fixed at the given time points and stained with H&E. Images represent magnification at 40X and 200X (inset) respectively. Age matched nulliparous *Ccnd1*$^{-/-}$ female mammary tissue serves as a negative control. D. H&E staining of the *Ccnd1*$^{-/-}$ retina of 4-week-old mouse demonstrating the retinal hypoplasia phenotype (Magnification 400X).

Figure S2. **Estimated left ventricular mass and diameter is altered in *Ccnd1*$^{KI/KI}$ animals.** A. *Ccnd1*$^{+/+}$ and *Ccnd1*$^{KI/KI}$ mice, 8-9 weeks of age, were monitored by echocardiogram for 1 hour. Cardiac function (A and B) as well as ventricular dimensions (C) are reported. n=3 mice per cohort. HR, heart rate; EF, ejection fraction; FS, fractional shortening; LVAW;d, end-diastolic left ventricular anterior wall; LVAW;s, end-systolic left ventricular anterior wall; LVID;d end-diastolic left ventricular internal dimension; LVID;s, end-systolic left ventricular internal dimension; LVPW;d, end-diastolic left ventricular posterior wall; LVPW;s, end-systolic left ventricular posterior wall; LV mass, left ventricular mass; LV Mass Corrected, left ventricular mass corrected for total body mass. Error bars represent the standard error of the mean (+/- SEM), and significance determined using a two-tailed students t-test. *=$p<0.05$, **=$p<0.01$

Figure S3. **Cyclin D1b expressing cells maintain +/+ levels of p-RB *in vivo* and cell cycle entry during serum deprivation.** A. The indicated tissue was harvested from age matched *Ccnd1*$^{+/+}$ and *Ccnd1*$^{KI/KI}$ animals, frozen in liquid nitrogen, and protein lysates generated using a

mortar and pestle in RIPA buffer. Levels of p-RB(S780) were determined via immuno-blot (n= at least 2/genotype). B. Indicated MAF lines were grown in full media for 24 hours at 60% confluency and lysed in D-Lysis buffer as previously described(Knudsen et al, 1998). Cells were incubated with CDK4, N-terminal Cyclin D1, or non-specific IgG antisera for 12 hours at 4C and immunoblotted for CDK4 expression. 20% input serves as a positive control for each cell line. C. Cells were plated as in C and allowed to grow for 48 hours. BrdU was added to cells for a period of 1 hour, after which cells were harvested for analyses via bivariate flow cytometry of active S-Phase (BrdU incorporation-bottom) and DNA content (propidium iodide-top). Quantification of biological triplicates is shown to the right. Error bars represent +/- standard error of the mean (+/- SEM) and significance determined using a two-tailed students t-test. ***=p<0.001

Figure S4. **Validation of intrinsic markers of DNA damage and PARP activity in an independently derived KI/KI MAF line.** A. Tissue was harvested from a 3-month-old *Ccnd1*$^{KI/KI}$ animal and utilized to generate a second stable murine adult fibroblast line (as in A). Passage matched cells were then grown in serum proficient media for 24 hours and stained for markers of DNA damage (p-H2AX and 53BP1). 3 random fields for each of 3 biological replicates was counted and data is presented as the % of cells with >10foci/cell (p-H2AX) or % of cells with >2 foci/cell (53BP1) (400x magnification). B. Cells were grown as in A, and then analyzed for PARP activity via immunoblot. Cyclin D1b and Lamin B serve as identity and loading controls respectively. Error bars represent +/- standard error of the mean (+/- SEM) and significance determined using ANOVA analyses. *=p<0.05

Figure S5. ***Ccnd1***$^{+/+}$ **and** ***Ccnd1***$^{KI/KI}$ **MAF's are p53 positive and induce the p53 target gene *Cdkn1a* in response to genomic stress**

A. *Ccnd1*$^{+/+}$ and *Ccnd1*$^{KI/KI}$ MAF lines were plated at equal density and grown in serum proficient media for 24 hours. Cells were then treated with either 0.5μM Etoposide or vehicle for 3 hours. After which, cells were harvested, RNA purified, cDNA generated, and relative expression of *Cdkn1a* transcript levels were determined via Q-RT-PCR. *Gapdh* transcript serves as a control. B. Cells were treated as in A and then immunoblotted for total p53 and p21. Lamin B serves as a loading control. Error bars represent +/- standard error of the mean (+/- SEM) and significance determined using a two-tailed students t-test.*=p<0.05, **=p<0.001

Figure S6. **Induction of Cyclin D1a is not associated with markers of DNA damage.** A. Isogenic lines expressing Cyclin D1a, Cyclin D1b, or Vec control in the LNCaP prostate cancer model were grown in serum proficient media for 24 hours, and then stained for the presence of p-H2AX and 53BP1 foci via immunofluorescence (400x magnification-left). 3 random fields from each of 3 biological replicates were counted and data is represented as the total number of foci/cell for each cell line. Error bars represent +/- standard error of the mean (+/- SEM) and significance determined using a ANOVA analyses.*=p<0.05, **=p<0.001
