# Supplementary material for: Consequence of the tumor-associated conversion to cyclin D1b
Source: EMBO Mol Med. 2015 Mar 18;7(5):628–47. doi: 10.15252/emmm.201404242 (PMC4492821; doi:10.15252/emmm.201404242)
Supplement: Supplementary file 2 [file emmm0007-0628-sd2.pdf]

Consequence of the tumor-associated conversion to Cyclin D1b

M. A. Augello, L.D.Berman-Booty, R. Carr 3rd, A. Yoshida, J.L.Dean, M.J.Schiewer, F.Y. Feng, S.A. Tomlins, E. Gao, W.J.Koch, J.L.Benovic, J.A. Diehl, and K.E. Knudsen

Corresponding author: Karen Knudsen, Kimmel Cancer Center/Thomas Jefferson University

Review timeline:

Submission date:	10 May 2014
Editorial Decision:	28 June 2014
Appeal:	07 July 2014
Additional editorial correspondence	07 July 2014
Additional author correspondence	10 July 2014
Additional editorial correspondence:	14 July 2014
Revision received:	18 December 2014
Editorial Decision:	21 January 2015
Revision received:	06 February 2015
Accepted:	13 February 2015

Transaction Report:

(Note: With the exception of the correction of typographical or spelling errors that could be a source of ambiguity, letters and reports are not edited. The original formatting of letters and referee reports may not be reflected in this compilation.)

Editor:

1st Editorial Decision 28 June 2014

Thank you for the submission of your manuscript to EMBO Molecular Medicine. We have now heard back from the three referees whom we asked to evaluate your manuscript.

We are very sorry that it has taken much longer than usual to get back to you on your manuscript. In this case we experienced significant difficulties in securing three expert and willing Reviewers. Further to this, one evaluation was delivered with considerable delay.

As you will see the Reviewers, in aggregate, point to significant and fundamental issues that, I am afraid, preclude publication of the manuscript in EMBO Molecular Medicine. I will not discuss each point in detail as they are clearly stated. There are, however, some points, in most cases shared by the Reviewers, that I wish to bring to your attention.

Reviewer 1, while more supportive of your study, is concerned that the knock-in mice do not spontaneously develop tumours, and although s/he admits that this is not without precedent, this is all the more reason to make sure that the in vitro studies deliver a clear message. This Reviewer also notes that data from human samples would be required to support the clinical relevance of the increased DNA damage in cyclin D1b overexpressing cells. S/he also lists several other items of concern that would need to be experimentally addressed.

Reviewer 2 also lists a number of critical points among which, for instance, that the experimental

approach based on the use of MAFs from the knock-in mice might be flawed. S/he also feels that the conclusion that cyclin D1b is a driver of damage is not supported.

Reviewer 3 is especially critical and suggests that the manuscript does not really provide significant new information on the function/action of cyclin D1b. S/he also laments the lack of comparison to cyclin D1 null mice, similarly to the other Reviewers, and the lack of in vivo tumourigenesis approaches.

When interrogated during the cross-commenting process, the Reviewers essentially agreed with each other and admitted that the amount of experimentation required was significant.

I am sure you will understand that all considered, the Reviewer concerns are too many and too fundamental and leave us no choice but to return the manuscript to you at this stage. In our assessment it is not realistic to expect to be able to address these issues experimentally, assuming of course the attainment of interesting results, in a reasonable time frame and to the satisfaction of the Reviewers.

I wish to add however that, considered the potential interest of these findings, we would have no objection to consider a new manuscript on the same topic if at some time in the near future you have obtained data that would considerably strengthen the message of the study and address the Reviewer concerns in full. Please do consider that if you were to submit a new manuscript this would be treated as a new submission, in particular with respect to the literature and the novelty of your findings at the time of resubmission, rather than a revision. If you decide to follow this route, please make sure you also submit a full rebuttal along with the manuscript.

At this stage of analysis, though, I am sorry to have to disappoint you. I hope that the Reviewer comments will be helpful in your continued work in this area.

***** Reviewer's comments *****

Referee #1 (Comments on Novelty/Model System):

Manuscript EMM-2014-04242

The manuscript by Augello and coworkers aims to characterize the oncogenic properties of a Cyclin D1 splicing variant (CycD1b) that is associated with tumor progression and poor clinical outcome in human patients. To this end the authors have generated a humanized version of CycD1b to assess the in vivo consequences of its expression under the control of the endogenous promoter. The authors report that CycD1b performs overlapping but non-redundant functions with wild type cyclin D1. The results also indicate that CycD1b performs oncogenic properties in vitro, provides novel links with the DNA damage machinery mediated by PARP1.
I find that the overall technical quality of the data presented in the manuscript is satisfactory and sufficiently supports the proposed claims. Yet I have seeveral concerns that need to be addressed.

1. My main concern regarding model the proposed by the authors is the lack of oncogenic properties in vivo. This is not without precedent, for instance a knock-in of K-RasG12V (no doubt a bona fide oncogene) is also tolerated by the vast majority of adult tissues in the mouse (Guerra et al, Cancer Cell 2003). Yet, since the putative oncogenic properties of CycD1b are based on the in vitro evidences these should be properly executed.
CycD1b adult murine adult fibroblasts (MAFs) are able to grow subcutaneously in nude mice and display atypia and invasion of the subcutaneous fat. This experiments provides two accepted hallmarks of oncogenicity. To complete the panel the authors should assess whether CycD1b MAFs also display anchorage independent growth. Also along these lines, do overexpressed CycD1a and D1b cooperate differently with single oncogenes in the transformation of primary MAFs? This could be performed with the oncogenes that have shown to require CycD1 such as K-Ras of Her2.

2. Another important point that is missing throughout the manuscript is a careful comparison of the expression levels of CycD1b and those of the wild type full length CycD1a. This is important since

the missing C-terminal part of the protein has been implicated in the control of CycD1 stability. Furthermore, expression levels are important for the transforming capacity, and even wild type CycD1 can be tumorigenic if overexpressed. There is not a single western blotting comparing the levels of CycD1 in wild type and CycD1b KI/KI cells or tissues in the same gel and using an antibody raised against a common epitope.

3. Following the same rationale of point 2, the 3¥-UTR of CycD1 has been shown to provide important regulatory functions. For instance several RNA binding proteins and miRNAs target this transcript and provide an important level or regulation. According to the diagram depicted in Fig 1A, the knock-in strategy is unlikely to affect the 3¥-UTR. The authors should at least comment this fact. Ideally, they should overexpress any of the various miRNAs that target this transcript and assess whether both the CycD1a and D1b transcripts behave similarly in vitro.

4. Another important point is to what extent the observed phenotypes are dependent on Cdk4/6 activity. Throughout the manuscript the authors suggest that the mot likely mechanisms is transcription dependent. This is of course possible but the authors should at least perform kinase assays to assess whether the Cyclin D1 associated kinase activity in CycD1b cells is increased compared to wild type controls.

5. The authors also report that Cyclin D1b expressing cells show increased DNA damage and are largely reliant on PARP1 signaling for the maintenance of genomic integrity. Furthermore they provide evidences that this fact could be exploited therapeutically. Overall this is an interesting observation.
I am aware of the difficulties in obtaining human samples, but it would be interesting to assess whether human tumor samples known to overexpress CycD1b also display increased gamma-H2AX foci.
In any case I am not completely convinced that what the authors describe are bona-fide double strand breaks. Overexpression of cyclins has also been associated with the induction of replicative stress. The authors need to further analyze the cellular response to rule out the presence of replicative stress by: i) performing 53BP1 foci in addition to gamma-H2AX staining ii) Perform a comet assay in CycD1a vs D1b cells iii) perform western blotting for phospho-Chk1 to investigate the presence of ATR activation (that would be associated with replicative stress).
Finally, the experiment shown in Fig5D with human prostate cells is not meaningful unless performed in parallel with overexpression of CycD1a in addition to the empty vector control.

Referee #2 (Remarks):

This manuscript describes the phenotype of mice and cells derived from them expressing exclusively the D1b form of cyclin D1 as a result of a humanized knockin construct. Previous work on D1b strongly supports its production and biological uniqueness in human tumors, and the alteration of the C-terminus of the protein as a result of alternative splicing produces significant functional and spatial changes when expressed in cells. This study advances the biological understanding of cyclin D1b in vivo and provides interesting evidence suggestive of heightened (and possibly unique) oncogenic role of this variant. Knockin mice are characterized for phenotypes characteristic of cylcin D1 knockout and kinase-dead knockin mice, and interesting similarities and disparities are noted. Importantly, 3T3 cells derived from these animals are transformed and show evidence of chronic DNA damage. Such transformed cells are sensitive to PARP inhibition, suggesting a method of attack on tumors that express D1b. In general this is an interesting study that could lead to significant clinical impact, but several issues need to clarified and/or expanded upon to achieve this impact:

1. A terminology quibble: the authors mention in several places, including very early in the discussion, that the D1b knocking does not "rescue" certain D1 knockout phenotypes, but does "rescue" others, such as retina formation. While there is no issue with the interpretation of these phenotypes, the use of "rescue" here gives the reader the wrong impression about how the studies were done and how the D1b protein might act. That is, it is more proper to say that D1b is deficient

for animal size control, survival, suppressing the clasping response, and for nursing pups (phenotypes present in knockout animals), but is proficient (retains functionality) in retinal development. This may seem semantic but I think better conveys the deficiency inherent in D1b when exclusively expressed in vivo.

2. The authors correctly note that the kinase-dead knockin mouse phenotype interestingly overlaps with D1b in the persistence of several phenotypes but lack of effect on the retina. (side note: to my knowledge the D1b heart development alteration was not reported in KO or KE animals as the authors suggest, but I'm not sure it's been specifically excluded, either. If not, this would suggest that it may be wholly accurate to call this a novel phenotype vis a vis D1 loss). However, I believe they miss the mark on the mammary gland/nursing phenotype. This is because the failure to nurse pups is likely due to at least two factors: one is lack of lobuloalveolar development in parous mice, easily seen by whole mount or H&E one day postpartum. The second is a lack of "husbandry" in that KO (and KE) females show no nesting response and thus failed lactation is likely to be only part of pups failure to thrive. Importantly, KE females do undergo lobuloalveolar development in the first pregnancy and appear to make milk, according to Landis' original report on the KE mouse.

Given the overlap between kinase dead and KI mice, and the difference from KO mice, it is important to discuss this issue of mammary development and to perform the above-mentioned histological analysis of mammary gland development.

3. Experiments in the latter half of the paper using 3T3s derived from MAFs are interesting, but suffer from a couple of ambiguities. First, because these experiments have not been done in parallel in KO MAFs (to my knowledge), it is hard to know if D1b behaves differently here from loss of D1(a). Second, how much of the transformed phenotype is the result of the immortalization process vs. intrinsic to MAFs? To address these issues, it would be ideal to make MAF-derived 3T3s from KOs, but this could be too extensive an experiment to be reasonable here, unless the authors already have them. It would therefore be sufficient to characterize primary MAFs for growth rate and serum deprivation response in comparison to WT primary MAFs, and to assess signs of DNA damage, prior to immortalization. It is also important to analyze RB phosphorylation in primary cells, as the persistence of S780 in 3T3s might be the consequence of selection for immortalization. This analysis of primary cells is crucial to support the authors' conclusions.

4. In keeping with 3 above, the conclusion that D1b drives damage, vs. acting as a null and impairing the DDR, as has been suggested for D1 KO, rests on speculation more than data. A simple way to support this concept better might be to knock down D1b in 3T3 cell lines and ask if they (1) lose their proliferative advantange in low serum (and RB phosphorylation) and (2) show reduced signs of persistent DSBs, like H2Ax positivity and PARP activation.

Referee #3 (Remarks):

The authors generated a mouse model for humanized Cyclin D1b, where Cyclin D1a is not expressed anymore. They go on to study the consequences of this change in living mice, which turn out to be viable. The data for the KI/KI mice is compared to the Cyclin D1-/- mice, although the latter is never shown. By this (virtual) comparison, some of the phenotypes in KI/KI are similar than KO, others are like wild-type. Overall, there is not dramatic phenotype described for the KI/KI mice and therefore it is difficult to get excited about this work. From this work, there is no new observation about possible mechanism of Cyclin D1b.
Nevertheless, the rational of generating these KI/KI mice is sound and interesting and the manuscript is well written. The results indicate that Cyclin D1b behaves to certain extent like wild-type Cyclin D1 but not always. It would be interesting to know why Cyclin D1b cannot fulfill the same functions like wild-type Cyclin D1.
The authors will need to try to make this manuscript more impactfull and need to address the following issues:
Major points:
1. The authors generated MAF (mouse adult fibroblasts) from the KI/KI mice. This seems a strange

© EMBO

choice since in most cases, MEFs (mouse embryonic fibroblasts) are generated from mice. The authors need to explain their choice and why MAF are better than MEFs. They are obviously aware that their results on MAF are difficult to compare to MEFs.
2. In Fig.3, 3T3 MAF form tumors when injected in nude mice. Although this experiments is fine, we don't learn too much from this. The authors would be much better of to use in vivo tumorigenesis approaches since they have generated the KI/KI mice. The results presented here are of limited value.
3. The authors study the growth behavior of MAFs and find that they are sensitive to low serum conditions. When they check BrdU incorporation, they find that there is no difference in the KI/KI between 10% and 1% serum but in wild-type MAFs, there is a decrease (as expected). How can the authors explain this contradictory results?
4. The expression of a number of cell cycle proteins are shown in Fig. 4. The authors should include a blot for total Cyclin D1 (any isoform) and need to look at the interaction with CDK4 and CDK6. Are the proteins binding to CDK4 and CDK6 the same in KI/KI in comparison to wild-type?
5. The authors treat MAFs with 5Gy IR and check BrdU incorporation in Fig.6. It seems that the decrease of BrdU incorporation in both wild-type and KI/KI MAFs is quite low at 5Gy IR. How can the authors explain this since these results differ completely what has been published before.

Appeal 07 July 2014

Thank you for sending such a thoughtful decision letter. If possible, I would like to discuss this with you by phone tomorrow. While we appreciated your concern, we respectful request the opportunity to revise the study without it being considered as a new manuscript. We feel that this is warranted, as:

1. The most impactful parts/major conclusions of the study (translational elements in figures 4 and beyond) were considered quite strong and raised no reviewer concerns
2. Essential elements of the critique for Figures 1-3 can be readily addressed in 2-3 months time. This includes the only comments (n=2) that were consistent across more than one reviewer.

We would be happy to provide a summation of the experiments already completed or in progress that will address the reviewer comments in full, as we have carefully assessed the concerns over the last few days. Please advise on a time that we can discuss! We have significant competition in this topic, and would like to resolve this as soon as possible.

Additional editorial correspondence 07 July 2014

Thank you for your message.

Unfortunately I am away at a meeting right now and will return in the office next Monday.

May I suggest the following course of action: please prepare a full point by point rebuttal addressing my and the Reviewers' concerns, which I will promptly discuss with my colleagues upon my return, after which I can discuss with you, over the phone if you wish. Do consider, however, that the relative importance of indicated issues is not determined by majority vote (i.e. how many Reviewers raise them)!

If you send in this rebuttal within the week, I will endeavor to deal with it soonest as I return.

Additional author correspondence 10 July 2014

Thank you for your prompt response and willingness to potentially consider a revision of our manuscript.

The study appears to have been well received by all reviewers, and would like to highlight that the most impactful and translation elements of the manuscript (centering on the consequence of tumor-associated Cyclin D1b and the utility of PARP1 inhibitors to target Cyclin D1b+ tumor cells) raised no reviewer concerns. With regard to the critiques, we generally agree with each reviewer concern, and as detailed below, are confident that we can address each concerns within a relatively short time frame (2-3 months). Below please find a detailed response to each of the reviewers concerns outlining how each point will be addressed in the revised manuscript.

Point-by-point-response:

1. Reviewer 1 comment 1-1: "*CycD1b adult murine adult fibroblasts (MAFs) are able to grow subcutaneously in nude mice and display atypia and invasion of the subcutaneous fat. This experiments provides two accepted hallmarks of oncogenicity. To complete the panel the authors should assess whether CycD1b MAFs also display anchorage independent growth.*

Response: We appreciate the comments of the reviewer and agree that demonstrating anchorage independent growth in models that harbor the switch to Cyclin D1b would further strengthen the conclusions and impact of the transformation study. As such, both +/+ and KI/KI MAF lines have been plated in soft agar and will be assed for anchorage independent growth. Based on precedent and the data already shown in the manuscript, we anticipate that KI/KI lines will display a marked ability to grow in soft agar, which will further support the stated conclusion that Cyclin D1b expression is associated with phenotypes related to transformation.

2. Reviewer 1 comment 1-2: *"…. do overexpressed CycD1a and D1b cooperate differently with single oncogenes in the transformation of primary MAFs? This could be performed with the oncogenes that have shown to require CycD1 such as K-Ras of Her2."* **Reviewer 3 comment 2:** *"In Fig.3, 3T3 MAF form tumors when injected in nude mice. Although this experiments is fine, we don't learn too much from this. The authors would be much better of to use in vivo tumorigenesis approaches since they have generated the KI/KI mice."*

Response: In the initial submission, we showed that while murine fibroblasts expressing endogenous Cyclin D1a are non-tumorigenic, engineering the CCND1 locus to express Cyclin D1b under the endogenous promoter resulted in transformation with 100% penetrance. However, in studies of up to 9 months, tumor incidence in the Cyclin D1b animals trended but was not significantly higher than that observed in wild-type. Therefore, it is quite sensible of the reviewer to ask if additional oncogenic insult can cooperate with Cyclin D1b to accelerate transformation. The reviewer astutely points out that transformation of primary cells is often enhanced by cooperation between deregulation of two or more oncogenes, and that it is indeed rare for a single genetic insult to induce tumorigenesis. The contribution of elevated levels of Cyclin D1a/Cyclin D1b alone or in combination with established oncogenes on transformation remained unclear. To address these concerns we plan to incorporate the following combination of existing data and new approaches:

1) *EXISTING DATA TO BE INCLUDED:* Early passage MAF models harboring either exogenic expression of either Cyclin D1a (in +/+ lines) or Cyclin D1b (in KI/KI lines) were already generated and assayed for tumor formation and growth in nude mice. In these head to head comparisons, Cyclin D1a was weakly tumorigenic, enhancing total tumor formation by less than 20%. While tumor incidence was similar in the Cyclin D1b+ cohort, tumors trended to form more quickly in the Cyclin D1b cohort. Thus, these data agree with the reviewer contention that a "second hit" is likely required for Cyclin D1b-induced tumor formation.

2) NEW APPROACHES: As suggested by the reviewer, both +/+ and KI/KI MAF lines will be engineered to stably express a constitutively active mutant of K-Ras (V12G). Isogenic pairs will then be plated in soft agar and assayed for growth after a period of 4 weeks. We anticipate that activated K-Ras will enhance the anchorage independent growth of both +/+ and KI/KI lines, but will cooperate with Cyclin D1b to enhance growth in this context. If time permits, cells will also be injected into nude mice to monitor tumor incidence and growth. These studies will therefore address the query of whether known oncogenes cooperate with Cyclin D1b to enhance tumor formation.

3. Reviewer 1 comment 2: *"Another important point that is missing throughout the manuscript is a careful comparison of the expression levels of CycD1b and those of the wild type full length CycD1a. This is important since the missing C-terminal part of the protein has been implicated in the control of CycD1 stability. Furthermore, expression levels are important for the transforming capacity, and even wild type CycD1 can be tumorigenic if overexpressed. There is not a single western blotting comparing the levels of CycD1 in wild type and CycD1b KI/KI cells or tissues in the same gel and using an antibody raised against a common epitope."*

Response: We thank the reviewer for pointing out this important experiment. To address these concerns we plan to execute the following experiments:

1) Analysis of mRNA levels of Cyclin D1a and Cyclin D1b in +/+ and KI/KI MAF lines using a primer pair common to both isoforms will be conducted in biological triplicate and analyzed by Taqman-based, quantifiable Q-PCR. Initial characterization of isoform expression in Figure 1C suggested that levels of *transcript a* and *transcript b* are comparable across tissue types, and as such we anticipate that these results will be recapitulated in the MAF model system.

2) Direct comparison of Cyclin D1a and Cyclin D1b levels will be determined in MAF model systems via immunoblot using an antibody directed against the N-terminus of the protein (e.g. the monoclonal antibody DCS-6 whose epitope is shared between the two isoforms). We anticipate comparable expression of the two isoforms based on previous reports which have characterized the stability of both proteins in human models. Interestingly, despite lacking the PEST domain which is required for efficient nuclear export and degradation of Cyclin D1a, the half life of Cyclin D1b was reported to be similar to that of Cyclin D1a (~30 minutes). Furthermore, evidence discussed above which analyzed tumor formation in response to elevated levels of Cyclin D1b found little effect on tumor incidence, collectively suggesting that the presence of Cyclin D1b rather than absolute levels of Cyclin D1b is largely responsible for its oncogenic properties.

4. Reviewer 1 comment 3: *"… the 3'UTR of CycD1 has been shown to provide important regulatory functions. According to the diagram depicted in Fig 1A, the knock-in strategy is unlikely to affect the 3'-UTR. The authors should at least comment this fact. Ideally, they should over-express any of the various miRNAs that target this transcript and assess whether both the CycD1a and D1b transcripts behave similarly in vitro."*

Response: We thank the reviewer for pointing out this interesting line of investigation. Unfortunately, the 3'UTR of the murine Cyclin D1 gene was also removed when generating the knock-in model. Preliminary data generated from human models of Cyclin D1b expression indicated that the 3'UTR of *transcript b* consisted of between 600-900 nucleotides encoded by intron 4, and largely lacked all sequences encoded by the terminal exon (exon 5) and the 3'UTR. Thus to mimic this structure as closely as possible, all of murine exon 4, intron 4, exon 5, and the 3'UTR were removed. Consequently, it is highly unlikely that *transcript b* is regulated by similar miRNA's as that of full-length *transcript a*. To clarify this point, Figure 1A will be modified to demonstrate removal of the 3'UTR of the *Ccnd1* gene, and specifically mentioned in the revised text.

5. Reviewer 1 comment 4: *"Another important point is to what extent the observed phenotypes are dependent on Cdk4/6 activity. Throughout the manuscript the authors suggest that the mot likely mechanisms is transcription dependent. This is of course possible but the authors should at least perform kinase assays to assess whether the Cyclin D1 associated kinase activity in CycD1b cells is increased compared to wild type controls."* And **Reviewer 3 comment 4:** *"The expression of a number of cell cycle proteins are shown in Fig. 4. The authors should look at the interaction with CDK4 and CDK6. Are the proteins binding to CDK4 and CDK6 the same in KI/KI in comparison to wild-type?"*

Response: The reviewer raises an excellent point. Previous reports have demonstrated that despite maintaining the ability to effectively bind to CDK4/6, Cyclin D1b is limited in its ability to promote CDK4/6 kinase activity and subsequent phosphorylation of RB (S780). However, these studies were performed in artificial, overexpression models, either in the presence of Cyclin D1a or in cells which had lost Cyclin D1 expression. Thus, to define the ability of endogenous Cyclin D1b to associate with and activate CDK4/6, kinase assays will be preformed in both KI/KI and +/+ lines and assayed for the presence of phosphorylated RB peptide, as per the reviewers suggestion. Notably, our new model system will the first to allow for such an important (and readily achievable) line of investigation.

6. Reviewer 1 comment 5-1: *"I am aware of the difficulties in obtaining human samples, but it would be interesting to assess whether human tumor samples known to overexpress CycD1b also display increased gamma-H2AX foci"*

Response: We agree with the reviewer and believe correlating Cyclin D1b expression to markers of genome instability in patient samples would greatly increase the clinical and translational impact of this study. As such, we have obtained human samples of castration resistant prostate cancer (n=~109) and have immuno-stained them for Cyclin D1b and PAR (which was found to be elevated in prostate cancer cells which express Cyclin D1b-Figure 5). These tissue microarray slides are currently being scored by a board certified pathologist for staining intensity and cellular distribution, and will be analyzed using correlation software. Based on the findings *in vitro* which uncovered markers of genome instability associated with Cyclin D1b expression across multiple model systems, we expect that tumors which express Cyclin D1b will demonstrate heighted PAR staining, laying foundation for Cyclin D1b to be developed as potential biomarker for a positive response to combined therapeutic intervention.

7. Reviewer 1 comment 5-2: *"I am not completely convinced that what the authors describe are bona-fide double strand breaks. Overexpression of cyclins has also been associated with the induction of replicative stress. The authors need to further analyze the cellular response to rule out the presence of replicative stress…"*

Response: To more fully define the cellular response seen in cells that express Cyclin D1b, both +/+ and KI/KI MAF lines will be assessed for the presence of intrinsic 53BP1 and y-H2AX foci. As per the reviewer's suggestion, measures of replicative stress will also be determined though assessment of p-Chk1 (active Chk1) via immunoblot. Collectively we anticipate that 53BP1 foci will correlate with the heightened y-H2AX foci seen in the KI/KI lines, providing an additional measure of genome instability in this model system. We are well versed in these readily achievable assays.

8. Reviewer 1 comment 5-3: *"Finally, the experiment shown in Fig5D with human prostate cells is not meaningful unless performed in parallel with overexpression of CycD1a in addition to the empty vector control."*

Response: We thank the reviewer for pointing out this critical control. Models system which have been engineered to over-express Cyclin D1a are currently in production, and isogenic pairs will be assayed for markers of DNA damage (e.g. y-H2AX foci and total PAR levels) in the LNCaP system.

9. Reviewer 2 comment 1: *"A terminology quibble: the authors mention in several places, including very early in the discussion, that the D1b knocking does not "rescue" certain D1 knockout phenotypes, but does "rescue" others, such as retina formation. While there is no issue with the interpretation of these phenotypes, the use of "rescue" here gives the reader the wrong impression about how the studies were done and how the D1b protein might act."*

Response: The reviewer points out that there could be confusion with potential readers regarding how the mouse studies were conducted if using the term "rescue" to reference phenotypes which were initially defined in the Cylin D1 $^{-/-}$ mouse. We appreciate such a distinction and will alter the text to more clearly articulate the phenotypes of the Cyclin D1 $^{KI/KI}$ animals

10. ***Reviewer 2 comment 2:*** *"I believe they miss the mark on the mammary gland/nursing phenotype. This is because the failure to nurse pups is likely due to at least two factors: one is lack of lobuloalveolar development in parous mice, easily seen by whole mount or H&E one day postpartum. Given the overlap between kinase dead and KI mice, and the difference from KO mice, it is important to discuss this issue of mammary development and to perform the above-mentioned histological analysis of mammary gland development."*

Response: We thank the reviewer for pointing out this distinction. We have already set up mating pairs for both *Ccnd1*$^{+/+}$ and *Ccnd1*$^{KI/KI}$ and will assess mammary gland branching 1 day postpartum via H&E as suggested by the reviewer.

11. ***Reviewer 2 comment 3:*** *"Experiments in the latter half of the paper using 3T3s derived from MAFs are interesting, but suffer from a couple of ambiguities. It would therefore be sufficient to characterize primary MAFs for growth rate and serum deprivation response in comparison to WT primary MAFs, and to assess signs of DNA damage, prior to immortalization. It is also important to analyze RB phosphorylation in primary cells, as the persistence of S780 in 3T3s might be the consequence of selection for immortalization. "*

Response: We appreciate the concerns of the reviewer, and have explored characterizing primary non-immortalized cells previously. Unfortunately, cells isolated in this manner undergo rapid cell cycle arrest or death and are often a mix of several cell types prior to immortalization. Consequently, it would be difficult to conclude much from such cell lines. However, the following experiments are underway to address these valid concerns.

1) Phosphorylation of RB will be assessed from several proliferative tissues of both +/+ and KI/KI mice, to demonstrate that p-RB S780 is preserved with Cyclin D1b expression. This data will be used in conjunction with the kinase assay discussed above to fully characterize the ability of Cyclin D1b to promote CDK4/6 activation in this context.

2) A second independently derived KI/KI MAF line will be used to demonstrate heightened y-H2AX foci formation as well as serum independent growth, providing further evidence to support the role of Cyclin D1b in promoting a state of genomic instability.

3) If time permits, Cyclin D1b will be stably introduced into +/+ MAF lines, and then assayed for both serum independent growth as well as genomic instability (also described below).

12. Reviewer 2 comment 4: *" …the conclusion that D1b drives damage, vs. acting as a null and impairing the DDR, as has been suggested for D1 KO, rests on speculation more than data. A*

simple way to support this concept better might be to knock down D1b in 3T3 cell lines and ask if they (1) lose their proliferative advantange in low serum (and RB phosphorylation) and (2) show reduced signs of persistent DSBs, like H2Ax positivity and PARP activation."

Response: To more completely define the function of Cyclin D1b in promoting both serum independent growth as well as genome instability we propose to:

1) Design siRNA which can target the humanized version of Cyclin D1b and assess the downstream effects on Rb phosphorylation, proliferative capacity, H2AX foci formation and PARP activity as suggested.

2) Induce Cyclin D1b expression in +/+ 3T3 models and determine the molecular impact on markers of both proliferative and genome instability phenotypes. Given the results generated from models mimicking Cyclin D1b expression in human cancer (Figure 4) we expect that introduction of Cyclin D1b into a +/+ background will phenocopy those demonstrated in the KI/KI lines.

13. Reviewer 3 comment 1: *"The authors generated MAF (mouse adult fibroblasts) from the KI/KI mice. This seems a strange choice since in most cases, MEFs (mouse embryonic fibroblasts) are generated from mice. The authors need to explain their choice and why MAF are better than MEFs."*

Response: While it is true that murine embryonic fibroblasts are used as models for a multitude of biological processes, murine adult fibroblasts (MAF's) provide an additional resource with which to study both developmental and tumorigenic phenotypes. Indeed many studies have utilized the MAF sysem to study how manipulation of tumor supressors or oncogenes contribute to transformation both *in vitro* and *in vivo*. Several of these studies also utilized human cancer lines to demonstrate that the effects observed in the MAF system could be recapitulated in human models (also demonstrated in our study-Figure 5). Thus, as we have significant expertise in generating these lines, and they have shown to be an effective model with which to study tumorigenic phenotypes, we chose to utilize MAF lines in leiu of their embryonic counterpart. **(**Buorgo et al 2011 Mol Cell), (de Napoles et al 2004 Developmental Cell), (Powers et al 2004, Mol Cancer Research) (Dean et al 2010, Oncogene).

15. Reviewer 3 comment 3: *"The authors study the growth behavior of MAFs and find that they are sensitive to low serum conditions. When they check BrdU incorporation, they find that there is no difference in the KI/KI between 10% and 1% serum but in wild-type MAFs, there is a decrease (as expected). How can the authors explain this contradictory results?"*

Response: This is an issue of misinterpretation, and we apologize for potential confusion in the initial text. The reviewer correctly points out that both +/+ and KI/KI lines demonstrate a reduced proliferative capacity in 1% as compared to full (10% serum) over time. However, the KI/KI lines show an enhanced ability to grow under such conditions (as compared to +/+ control), consistent with a transformed phenotype. To more completely define the acute cell cycle profile under these conditions, both +/+ and KI/KI models were analyzed for BrdU incorporation after 24 hours incubation in 1% serum. Consistent with the growth curve data at this time point, KI/KI lines maintain the capacity to grow with kinetics that mimic full serum, while there is a dramatic and sudden cell cycle arrest in the +/+ control line. As these experiments were designed to define the immediate response to 1% serum, extended time points were not included in this study. However, we anticipate that by 48 hours there would be a reduction in the KI/KI line consistent with the slightly lower proliferative rate observed in the growth curve described above. We again apologize for the confusion and will rigorously modify the text in the revised manuscript to clearly articulate this important point.

***16. Reviewer 3 comment 5:** "The authors treat MAFs with 5Gy IR and check BrdU incorporation in Fig.6. It seems that the decrease of BrdU incorporation in both wild-type and KI/KI MAFs is quite low at 5Gy IR. How can the authors explain this since these results differ completely what has been published before."*

Response: Again, this is an unfortunate issue of misinterpretation that requires clarification in the text. Both the BrdU incorporation assays as well as the B-galactosidase measurements were conducted 48 hours post treatment. As such, the cells had recovered from the initial insult. These timepoints were chosen so as to define the ability of both the +/+ and KI/KI lines to recover after DNA damage. However, the reviewer appears to be suggesting that inclusion of an earlier time point may also be of benefit, so as assess relative checkpoint in response to IR. This is readily achievable. As such, +/+ and KI/KI MAF models will be treated as in Figure 6 and assayed for BrdU incorporation 6 hours post treatment, to define the checkpoint response in the presence and absence of Cyclin D1b. Based on preliminary data which suggested that both +/+ and KI/KI lines resolve H2AX foci at comparable rates post IR, we anticipate that both lines will arrest with similar kinetics post treatment.

Additional editorial correspondence 14 July 2014

Thank you for your letter, rebuttal and suggested revision outline. I have now had the opportunity to carefully read them and I have also discussed them with my colleagues.

Considered your points and after discussion, we would be prepared to consider a substantially revised submission, with the understanding that the Reviewers' concerns must be addressed as per your outline with additional experimental data where appropriate and that acceptance of the manuscript will entail a second round of review. I will thus revert our editorial decision to "Revise and Re-Review" on our system to allow submission of your revised manuscript in due time.

Please note that it is EMBO Molecular Medicine policy to allow a single round of revision only and that, therefore, acceptance or rejection of the manuscript will depend on the completeness of your responses included in the next, final version of the manuscript.

As you know, EMBO Molecular Medicine has a "scooping protection" policy, whereby similar findings that are published by others during review or revision are not a criterion for rejection.

However, I do ask you to get in touch with us after three months if you have not completed your revision, to update us on the status. Please also contact us as Editorial decision to soon as possible if similar work is published elsewhere.

We look forward to receiving your revised mansucript.

1st Revision - authors' response 18 December 2014

Thank you for considering our manuscript for revision.

We are pleased that the study was well received by the reviewers. Notably, the most impactful and translational elements of the manuscript (centering on the consequence of tumor associated Cyclin D1b and the utility of PARP1 inhibitors to target Cyclin D1b+ tumor cells) raised no reviewer concerns. Rather, concerns centered on a subset of experimental details and requests for extensions of the findings. As detailed below, we have effectively addressed each reviewer concern. Below

please find a detailed response to each of the reviewers concerns outlining how each point was addressed in the revised manuscript.

Point-by-point-response:

1.Reviewer 1 comment 1-1: *"CycD1b adult murine adult fibroblasts (MAFs) are able to grow subcutaneously in nude mice and display atypia and invasion of the subcutaneous fat. This experiments provides two accepted hallmarks of oncogenicity. To complete the panel the authors should assess whether CycD1b MAFs also display anchorage independent growth."*

Response: We appreciate the comments of the reviewer and agree that demonstrating anchorage independent growth in models that harbor the switch to Cyclin D1b would further strengthen the conclusions and impact of the transformation study. As such, both +/+ and KI/KI MAF lines were plated in soft agar and assessed for anchorage independent growth. Consistent with tumor data presented in the initial manuscript, +/+ MAFs were incapable of growing in soft agar over a period of 3 weeks (**Modified Figure 4**). Conversely, KI/KI lines effectively formed colonies by 3 weeks, which provides further evidence to suggest that Cyclin D1b harbors oncogenic functions.

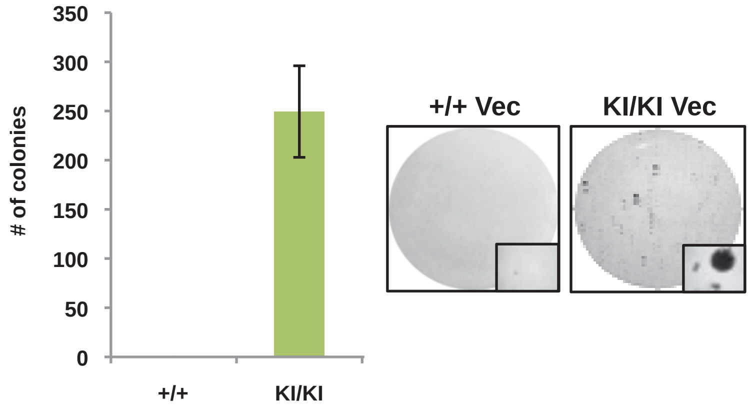

Modified from Revised Figure 4. Induction of Cyclin D1b promotes anchorage independent growth. Passage-matched *Ccnd1*$^{+/+}$ and *Ccnd1*$^{KI/KI}$ cells were mixed with 0.6% agar and allowed to grow for a period of 3 weeks. Cells were then fixed, stained with 0.01% crystal violet, and total number of colonies greater than 50µm were counted. Graph represents the average colony number of 6 biological replicates for each genotype

2. Reviewer 1 comment 1-2: *"…. do overexpressed CycD1a and D1b cooperate differently with single oncogenes in the transformation of primary MAFs? This could be performed with the oncogenes that have shown to require CycD1 such as K-Ras of Her2." and* **Reviewer 3 comment 2:** *"In Fig.3, 3T3 MAF form tumors when injected in nude mice. Although this experiments is fine, we don't learn too much from this. The authors would be much better of to use in vivo tumorigenesis approaches since they have generated the KI/KI mice."*

Response: In the initial submission, we showed that while murine fibroblasts expressing endogenous Cyclin D1a are non-tumorigenic, engineering the Ccnd1 locus to express Cyclin D1b under the endogenous promoter resulted in transformation with 100% penetrance. However, in studies of up to 9 months, tumor incidence in the Cyclin D1b animals trended but was not significantly higher than that observed in wildtype. Therefore, it is reasonable to ask if additional oncogenic insult can cooperate with Cyclin D1b to accelerate transformation. The reviewer astutely points out that transformation of primary cells is often enhanced by cooperation between deregulation of two or more oncogenes, and that it is indeed rare for a single genetic insult to induce

tumorigenesis. The contribution of elevated levels of Cyclin D1a/Cyclin D1b alone or in combination with established oncogenes on transformation remained unclear. To address these concerns we have completed the following studies:

1) Early passage MAF models harboring exogenous expression of either Cyclin D1a (in +/+ lines) or Cyclin D1b (in KI/KI lines) were generated (**Revised Figure 3E**) and assayed for tumor formation and growth in nude mice (**Revised Figure 3D-G**). In these head to head comparisons, elevated Cyclin D1a was weakly tumorigenic, enhancing total tumor formation by less than 25%. While tumor incidence was similar in the Cyclin D1b+ cohort, tumors trended to form more quickly in the Cyclin D1b high cohort. Thus, these data agree with the reviewer contention that a "second hit" is likely required for Cyclin D1b-induced tumor formation.

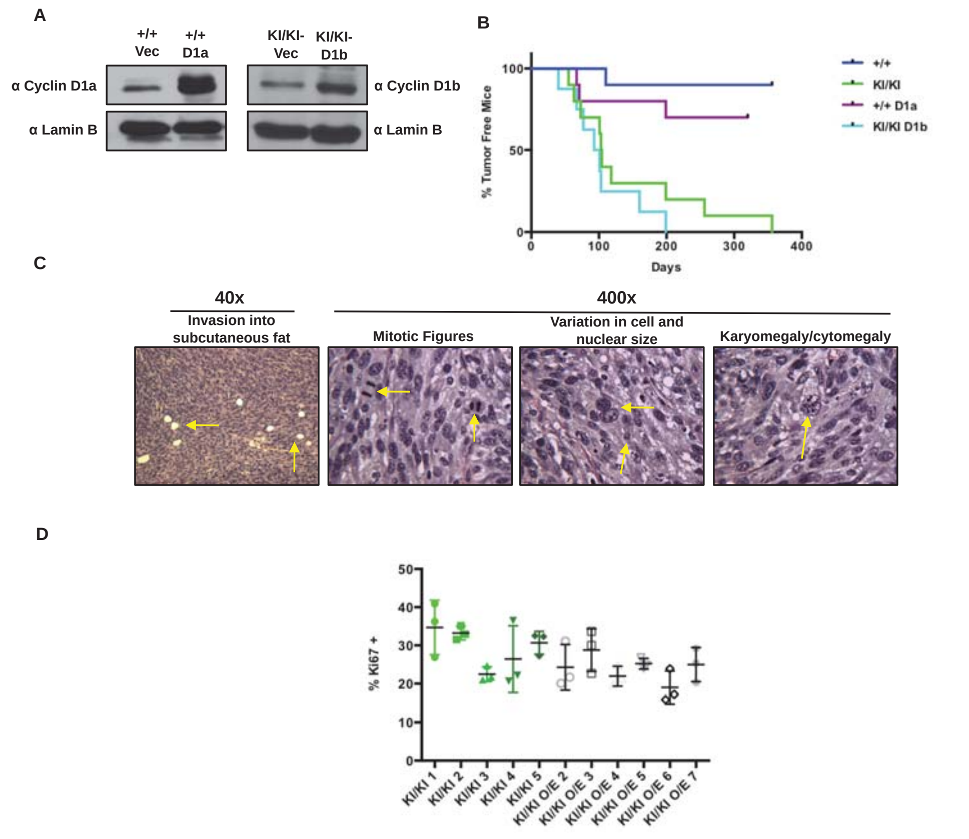

Modified from Revised Figure 3: Elevated expression is not required for Cyclin D1b mediated tumor growth *in vivo*. A. Immunoblot of stable induction of Cyclin D1a (in +/+ lines) or Cyclin D1b (KI/KI lines) and respective vector control. B. Cells described in A were subcutaneously implanted into the flanks and nude mice and monitored for tumor formation. Data is plotted as % of tumor free mice/time. C. Histological characterization of tumor associated characteristics of tumors formed from KI/KI-O/E cells. D. Comparison of Ki67 positivity between KI/KI-vec and KI/KI-O/E tumors plotted as percent of cells positive for KI67. Average Ki67 score is plotted for individual tumors.

© EMBO

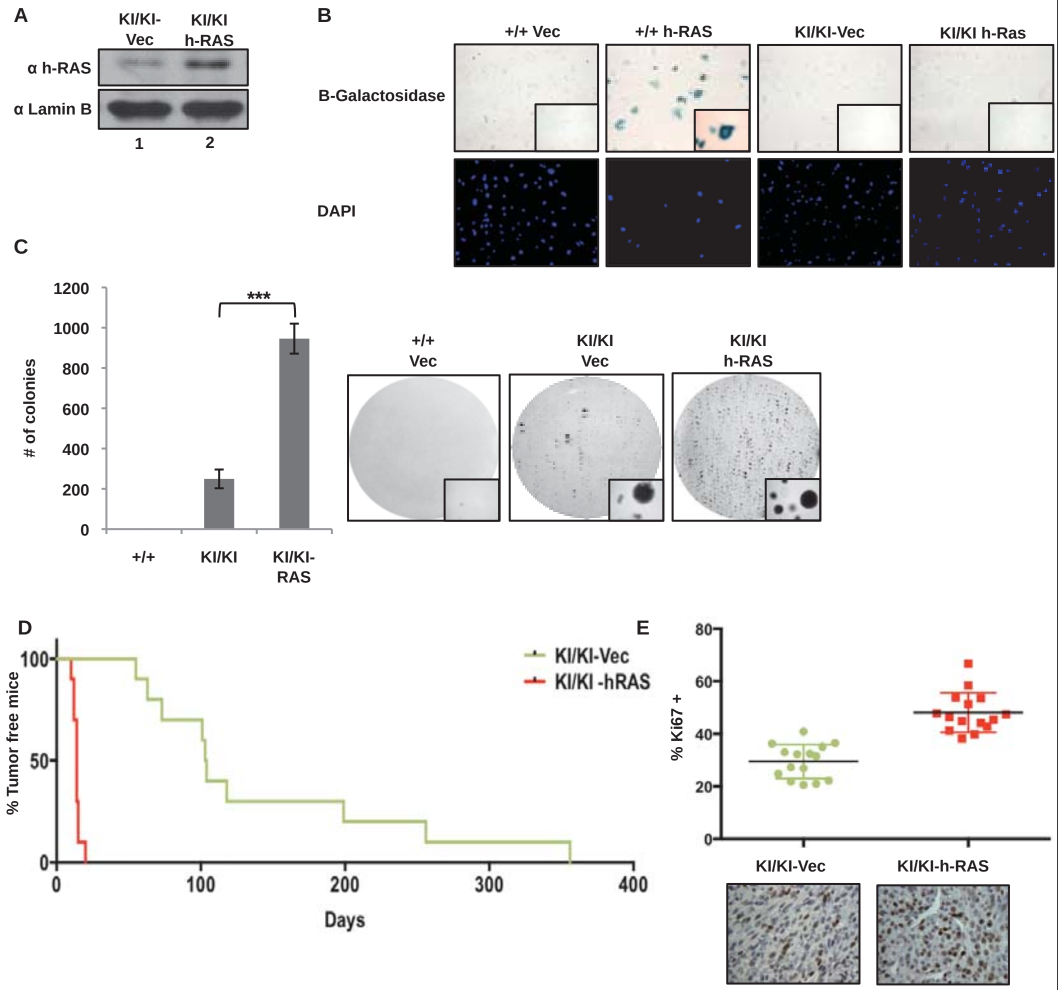

Revised Figure 4: Cyclin D1b cooperates with known oncogenes to foster tumor growth *in vivo*. A. +/+ and KI/KI lines were infected with vector or h-RAS expressing lentivirus and stable populations were generated via puromycin selection. Immunoblot demonstrates induction of h-RAS compared to control in KI/KI lines. B. Beta-Galactasidase activity of +/+ and KI/KI lines 7 days after infection with ctrl or h-Ras virus described in A. Blue color indicates positive staining and Dapi serves as a cell number control. C. +/+-vec, KI/KI-vec and KI/KI-Ras cells were plated in soft agar and allowed to grow for 3 weeks. After which, colonies greater than 50µm were counted and plotted as total colonies/plate. D. KI/KI-vec and KI/KI-Ras were subcutaneously injected into the flanks of nude mice and monitored for tumor formation. Data is plotted as % Ki67 +/total cell number.

2) As suggested by the reviewer, both +/+ and KI/KI MAF lines were infected with a construct to induce expression of h-RAS (**Revised Figure 4A**). As expected, based on the literature (reviewed in David G, et al. Curr Cancer Drug Targets, 2010), h-RAS in +/+ lines drove cells into a senescent state, whereas no such effect was seen in Vec control +/+ lines **Revised Figure 4B**. By contrast, KI/KI lines were resistant to oncogene-induced senescence and stable cell lines were readily formed. Furthermore, it was determined that Ras functioned to enhance the oncogenic functions of Cyclin D1b expressing cells, dramatically increasing growth in soft agar, and tumor growth *in vivo* **(Revised Figure 4C and D**). Collectively these data further implicate Cyclin D1b induction as a critical oncogenic “hit” which facilitates accumulation of additional oncogenic events to drive tumor formation and progression.

3. Reviewer 1 comment 2: *“Another important point that is missing throughout the manuscript is a careful comparison of the expression levels of CycD1b and those of the wild type full length*

CycD1a. This is important since the missing C-terminal part of the protein has been implicated in the control of CycD1 stability. Furthermore, expression levels are important for the transforming capacity, and even wild type CycD1 can be tumorigenic if overexpressed. There is not a single western blotting comparing the levels of CycD1 in wild type and CycD1b KI/KI cells or tissues in the same gel and using an antibody raised against a common epitope."

Response: We thank the reviewer for pointing out this important experiment and have addressed these concerns as follows:

First, analysis of mRNA levels of Cyclin D1a and Cyclin D1b in +/+ and KI/KI MAF lines using a primer pair common to both isoforms was conducted in biological triplicate and analyzed by Taqman-based, quantifiable Q-PCR. Interestingly, levels of transcript b (in KI/KI lines) was significantly lower than that of full length Cyclin D1a (in +/+ cells) (**Revised Figure 3B)**. Secondly, this difference was also observed at the protein level where direct comparison of Cyclin D1a and Cyclin D1b levels was determined via immunoblot using an antibody directed against the N-terminus of the protein (**Revised Figure 3B)**. At both the RNA and protein level, Cyclin D1b was approximately four fold lower than that of Cyclin D1a, suggesting that regulation of Cyclin D1b transcript rather than Cyclin D1b protein is responsible for the observed difference. This postulate was confirmed via analysis of Cyclin D1 transcript stability, wherein a significant decrease of transcript b was noted as early as 1 hour post treatment with the transcriptional inhibitor Actinomycin D. This trend continued over the course of 3 hours, whereas there was no significant difference in the abundance of transcript a in +/+ in this time frame. These data thus provide evidence to suggest that transcript b is less stable than that of transcript A in MAF models, and likely underlies the differences noted in protein abundance. Importantly data provided above which exogenously induces Cyclin D1b expression in KI/KI lines (**Revised Figure 3)** suggests that heightened levels of Cyclin D1b are not required for its oncogenic functions, and provide further evidence to suggest that the expression of Cyclin D1b, rather than absolute levels is important for its role as an oncogene.

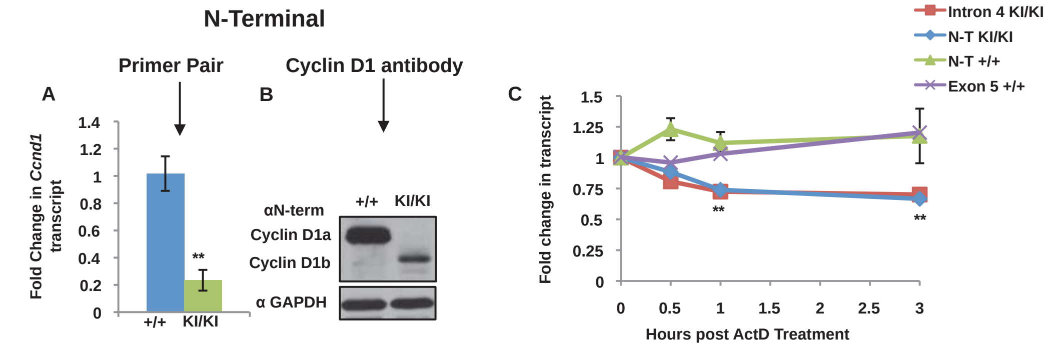

Modified from Revised Figure 3: Cyclin D1b is expressed at lower levels than that of Cyclin D1a in MAF models. A. Expression of the *Ccnd1* transcript in passage matched +/+ and KI/KI MAF lines using a primer pair targeted against the *Ccnd1* N-terminus (common to both isoforms). *Ccnd1* transcript levels are normalized to +/+ control. B. Cells described in A were probed for Cyclin D1 expression using an antibody directed against the N-terminus of Cyclin D1. GAPDH serves as a control. C. Cells in A were grown in full serum for 24 hours, then treated with transcriptional inhibitor Actinomycin D (0.25mM) for the time points indicated. RNA was purified from each cell line in biological triplicate and transcript level determined by Q RT-PCR. Primer sets unique to transcript a (Exon 5), unique to transcript b (Intron 4), or common to both isoforms (N-T) were utilized to define the abundance of the Cyclin D1 transcript for each genotype. ** p<0.01

4. Reviewer 1 comment 3: *"… the 3'UTR of CycD1 has been shown to provide important regulatory functions. According to the diagram depicted in Fig 1A, the knock-in strategy is unlikely to affect the 3'-UTR. The authors should at least comment this fact. Ideally, they should over-express any of the various miRNAs that target this transcript and assess whether both the CycD1a and D1b transcripts behave similarly in vitro."*

© EMBO

Response: We thank the reviewer for raising this point for discussion. As, is now more clearly depicted in **Revised Figure 1A**, the 3'UTR of the murine Cyclin D1 gene was also removed when generating the knock-in model. Preliminary data generated from human models of Cyclin D1b expression indicated that the 3'UTR of *transcript b* consisted of between 600-900 nucleotides encoded by intron 4, and largely lacked all sequences encoded by the terminal exon (exon 5) and the 3'UTR. Thus to mimic this structure as closely as possible, all of murine exon 4, intron 4, exon 5, and the 3'UTR were removed. As such, miRNA regulation is likely to be distinct.

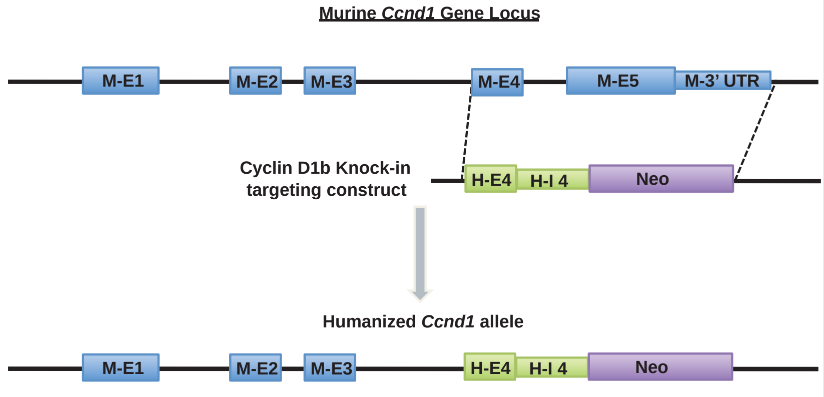

Revised Figure 1A: Humanization of the murine *Ccnd1* locus results in expression of Cyclin D1b with a unique C-terminus . Schematic of the murine *Ccnd1* gene locus and genetic strategy used to humanize the locus to produce Cyclin D1b. Note, sequences encoding murine exon 4, intron 4, exon 5, and 3' UTR were removed to force expression of human Cyclin D1b.

5. Reviewer 1 comment 4: *"Another important point is to what extent the observed phenotypes are dependent on Cdk4/6 activity. Throughout the manuscript the authors suggest that the mot likely mechanisms is transcription dependent. This is of course possible but the authors should at least perform kinase assays to assess whether the Cyclin D1 associated kinase activity in CycD1b cells is increased compared to wild type controls."* And **Reviewer 3 comment 4:** *"The expression of a number of cell cycle proteins are shown in Fig. 4. The authors should look at the interaction with CDK4 and CDK6. Are the proteins binding to CDK4 and CDK6 the same in KI/KI in comparison to wild-type?"*

Response: The reviewer raises an excellent point. As is shown in **Revised Figures 5C and E3B**, antisera directed against CDK4 or the N-terminus of Cyclin D1 efficiently co-precipitates with CDK4, consistent with previous reports which demonstrate that Cyclin D1a and Cyclin D1b bind to CDK4 with similar affinity (Lu et al, 2003 Cancer Res). Interestingly, analysis of CDK4 kinase activity (immunoprecipitated from +/+ and KI/KI cells-**Revised Figures 5C)** utilizing full length RB as a substrate, demonstrated nearly identical capacity to phosphorylate RB **i**n this context **(Revised Figures 5C).** Given that the phenotypes of the Cyclin D1b knockin mouse closely mimic those reported in other murine models which harbor a mutant allele of Cyclin D1 which is unable to activate CDK4/6 kinase activity, it is likely that immortalized cells expressing Cyclin D1b utilize additional, parallel pathways to activate CDK4 activity and drive cell cycle entry. This concept is supported by data presented above which demonstrated only ~20% reduction in cell cycle kinetics after efficient knockdown of Cyclin D1b (as compared to ~80% reduction in +/+ cell cycle kinetics after loss of Cyclin D1a). Collectively, these data are consistent with previous findings that suggest that Cyclin D1b is a poor activator of CDK4 kinase activity (Solomon DA et. al. 2003 JBC), and

suggest that cells expressing Cyclin D1b likely utilize alternative pathways to active CDK4/6 activity.

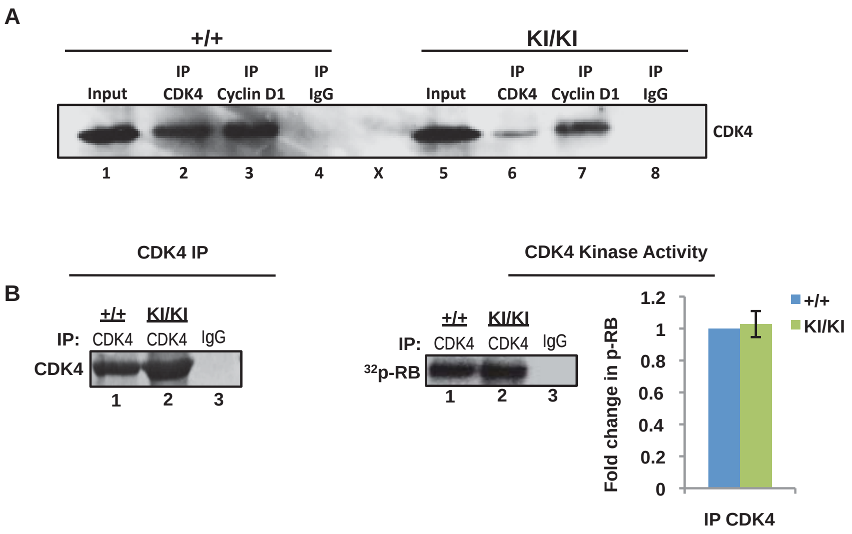

Modified from Revised Figures 5 and E3: CDK4 activity is comparable between cells expressing Cyclin D1a and Cyclin D1b: A. Indicated MAF lines were grown in complete media and immunoprecipitated with antisera specific to CDK4, the N-Terminus of Cyclin D1, or non-specific immunoglobulins. Samples were precipitated using Protein A conjugated beads and analyzed via immunoblot for the presence of CDK4. 20% Input serves as a positive control. B. Cells were immunoprecipitated for CDK4 as in A, and then incubated with purified full-length RB and ^{32}P-ATP for 30 minutes. Radioactivity for each sample was quantified and normalized to +/+ control (Right) and efficiency of CDK4 pulldown (Left). Data represents the average of 3 independent biological replicates.

6. Reviewer 1 comment 5-1: *"I am aware of the difficulties in obtaining human samples, but it would be interesting to assess whether human tumor samples known to overexpress CycD1b also display increased gamma-H2AX foci"* and ***Reviewer 1 comment 5-3:*** *"Finally, the experiment shown in Fig5D with human prostate cells is not meaningful unless performed in parallel with overexpression of CycD1a in addition to the empty vector control.*

*"***Response:** We agree with the reviewer and believe correlating Cyclin D1b expression to markers of genome instability in patient samples would be of significant value. Unfortunately, despite multiple attempts and utilizing several different protocols, we were unable to reliably detect and quantify p-H2AX foci in clinical samples by immuno-fluorescence. Difficulty in this process was confirmed by our co-author (Dr. Felix Feng) who is an expert radiation oncologist. It was suggested that the process of fixation, paraffin embedding, or a combination of both either destroys or masks the epitopes required for effective detection of p-H2AX, and currently there is no clinically validated protocol which can be used to define either p-H2AX or 53BP1 foci to help direct therapeutic care. Thus, to define the relevance of this pathway in human disease models of prostate cancer (which are known to induce Cyclin D1b expression at high frequency) were engineered to ectopically express vector control, Cyclin D1a, and Cyclin D1b and stained for intrinsic p-H2AX and 53BP1 foci **(Revised Figures 6E and E6)**. Interestingly, induction of Cyclin D1a had little effect on the presence of p-H2AX or 53BP1 foci, consistent with the proposed role of Cyclin D1a as a tumor suppressor in this tumor type. Inversely, and consistent with the posit that Cyclin D1b destabilizes genomic integrity, induction of Cyclin D1b was uniquely associated with significantly

elevated numbers of both 53BP1 and p-H2AX foci/cell **(Revised Figures 6E and E6)**, providing further evidence to demonstrate that Cyclin D1b expression is associated with heightened presence of DNA damage markers. Collectively, these findings are the first to demonstrate across multiple murine and human models that Cyclin D1b acts to maintain a state of genomic instability, which likely underlies the oncogenic functions of this Cyclin D1 isoform.

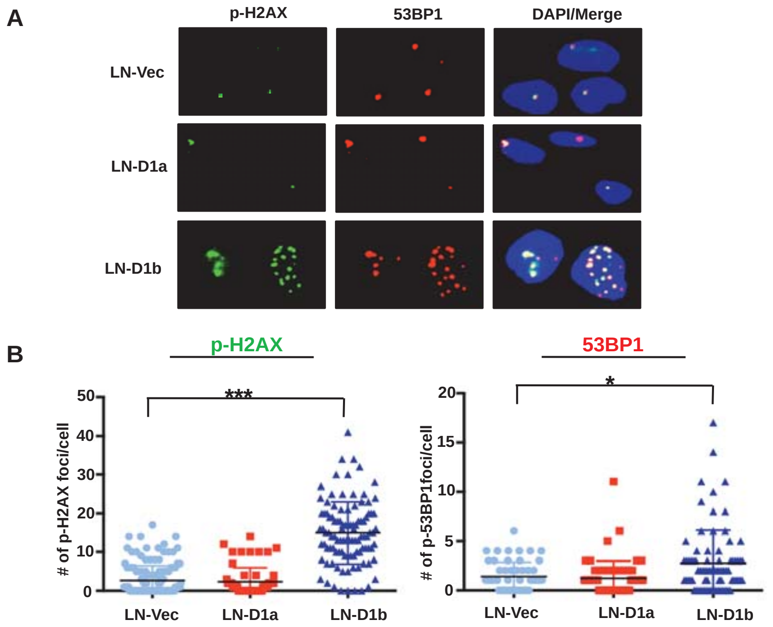

Modified from Revised Figure E6: Cyclin D1b induction is associated with markers of genome instability in models of prostate cancer. A. LNCaP prostate cancer cells were induced to express Cyclin D1a or Cyclin D1b or vector control and co-stained for markers of DNA damage (p-H2AX and 53BP1). DAPI serves as a nuclear control. B. Quantification of the images from A. 3 random fields from each of three biological replicates were quantified for the total number of foci present /cell. * p>0.05, ***p>0.001

***7. Reviewer 1 comment 5-2: "**I am not completely convinced that what the authors describe are bona-fide double strand breaks. Overexpression of cyclins has also been associated with the induction of replicative stress. The authors need to further analyze the cellular response to rule out the presence of replicative stress…" and**. Reviewer 2 comment 4:** "…the conclusion that D1b drives damage, vs. acting as a null and impairing the DDR, as has been suggested for D1 KO, rests on speculation more than data. A simple way to support this concept better might be to knock down D1b in 3T3 cell lines and ask if they (1) lose their proliferative advantage in low serum (and RB phosphorylation) and (2) show reduced signs of persistent DSBs, like H2Ax positivity and PARP activation."*

Response: To more completely define the function of Cyclin D1b in promoting both serum independent growth as well as genome instability a validated pool of 4 different siRNA's targeted against the N-terminus of the *Ccnd1* murine transcript or scramble control were transfected into both +/+ and KI/KI MAF lines and assessed for serum independent growth as well as p-H2AX and 53BP1 foci.

1) *Serum independent growth:* As is shown in **Revised Figures 5 and 6**, introduction of siRNA directed against Cyclin D1 resulted in a dramatic reduction of Cyclin D1a (Left) in +/+ cells, as well as Cyclin D1b (Right) in KI/KI cells. In full serum conditions, loss of Cyclin D1 correlated with a

dramatic reduction in the proliferative markers p-RB (S780) and Cyclin A, and was associated with a substantial decrease in BrdU incorporation (by ~82%) at 48 hours (**Revised Figures 5E-F**). Interestingly under identical conditions, loss of Cyclin D1b expression resulted in only a modest reduction in p-RB (S780) and Cyclin A levels, and reduced BrdU incorporation by only 22%. These data demonstrate that Cyclin D1b is largely dispensable for proliferation of KI/KI lines under full serum conditions. Inversely, loss of Cyclin D1b was found to be required for proliferation under serum starved conditions (**Revised Figures 5F)**. While loss of Cyclin D1a had no effect on the proliferative capacity of +/+ lines after 48 hours in 1% serum, reduction in Cyclin D1b dramatically reduced BrdU incorporation of KI/KI lines under similar conditions (**Revised Figures 5F**). Collectively, these data highlight the requirement for Cyclin D1b expression in promoting serum independent growth, and suggest that Cyclin D1b acts in an oncogenic capacity (rather than as a null allele) to regulate growth in this context.

2) *Markers of DNA Damage:* Utilizing similar time points as above, markers of genome stability were assed in both +/+ and KI/KI MAF lines after knockdown of Cyclin D1 isoforms. Consistent with what was observed previously, KI/KI lines harbor a greater number of cells with elevated p-H2AX and 53BP1 foci in control conditions (**Revised Figures 6A**). Importantly, loss of Cyclin D1b (in KI/KI lines) resulted in a reduction in the prevalence of both markers, as well as in reduced levels of PAR (**Revised Figures 6A and D**) suggesting that Cyclin D1b plays an active role in regulating genomic maintenance. These data together implicate Cyclin D1b as a driver of oncogenic phenotypes, and provide further evidence to link this isoform in the deregulation of both cell cycle and DNA damage pathways.

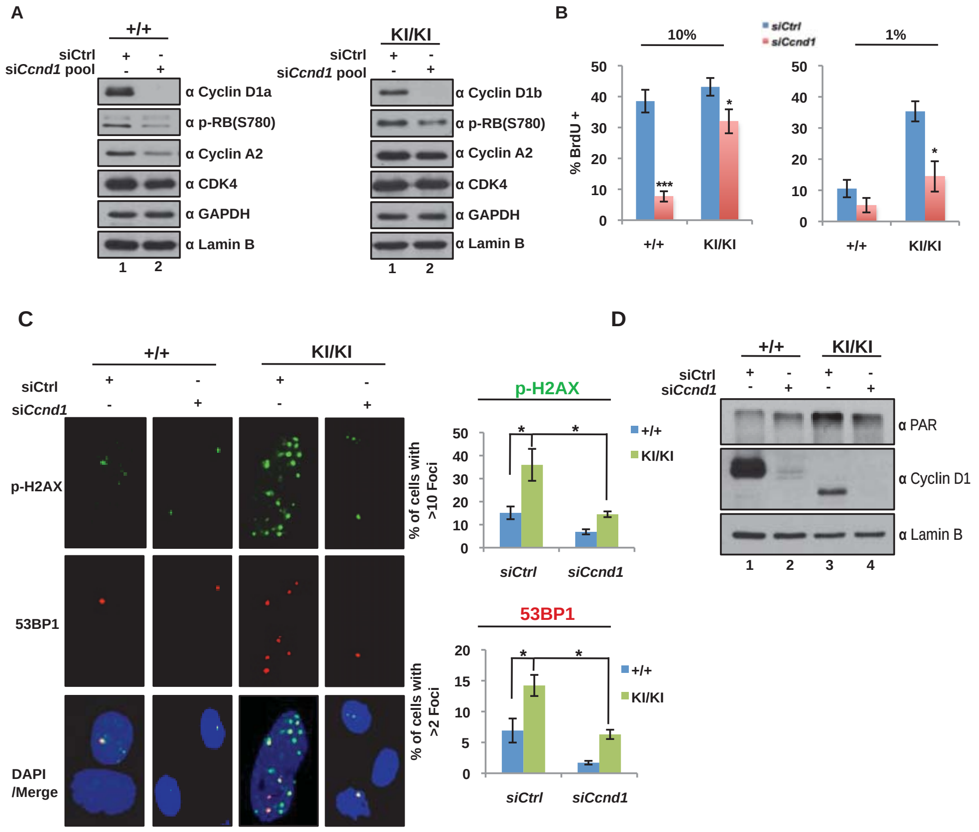

Modified from Revised Figures 5 and 6: Loss of Cyclin D1b inhibits serum independent growth and reduces markers of intrinsic DNA damage. A. A pool of validated siRNA directed against the N-terminus of murine Cyclin D1 or scrambled control were transfected into passaged matched +/+ and KI/KI MAF lines and probed for expression of cell cycle markers via immunoblot 48 hours post transfection. Lamin B and GAPDH serve as loading controls. B. Cells were treated as in A, treated with BrdU for 1 hr, and then assessed for BrdU positivity via immunofluorescence. Graphs represent % of positive cells/total cell population. At least 3 random fields from each of three biological replicates was counted for each condition. C. Cells were treated as in A and stained for the presence of p-H2AX (green), 53BP1 (red), and DAPI (blue). Graph represents the population of cells relative to total cell number which harbored >9 foci/cell (p-H2AX) and >2 foci/cell (53BP1). At least 3 random fields from each of three biological replicates were counted. D. Cells were treated as in A and immunoblotted for the presence of PAR and Cyclin D1 isoforms. Lamin B serves as a loading control * p<0.05, ** p<0.01

*9. **Reviewer 2 comment 1:** "A terminology quibble: the authors mention in several places, including very early in the discussion, that the D1b knocking does not "rescue" certain D1 knockout phenotypes, but does "rescue" others, such as retina formation. While there is no issue with the interpretation of these phenotypes, the use of "rescue" here gives the reader the wrong impression about how the studies were done and how the D1b protein might act."*

Response: The reviewer points out that there could be confusion with potential readers regarding how the mouse studies were conducted if using the term "rescue" to reference phenotypes which were initially defined in the Cyclin D1 $^{-/-}$ mouse. We appreciate such a distinction and have altered the manuscript throughout to more clearly articulate the observed phenotypes of the Cyclin D1 $^{KI/KI}$ animals.

*10. **Reviewer 2 comment 2:** "I believe they miss the mark on the mammary gland/nursing phenotype. This is because the failure to nurse pups is likely due to at least two factors: one is lack of lobuloalveolar development in parous mice, easily seen by whole mount or H&E one day postpartum. Given the overlap between kinase dead and KI mice, and the difference from KO mice, it is important to discuss this issue of mammary development and to perform the above-mentioned histological analysis of mammary gland development."*

Response: We thank the reviewer for pointing out this distinction. As suggested mammary tissue from *Ccnd1*$^{+/+}$ and *Ccnd1*$^{KI/KI}$ age matched females was analyzed for glandular branching and development in response to pregnancy. Interestingly, analysis of tissue sections by a board certified pathologist found no differences between *Ccnd1*$^{+/+}$ and *Ccnd1*$^{KI/KI}$ females post pregnancy (**Revised Figure E1C).** In both genotypes, extensive branching was noted across multiple glands, and contained secretions that were consistent with milk. As the reviewer pointed out, these data are consistent previous kinase dead knockin models of Cyclin D1 which indicated that the kinase function of Cyclin D1 is not required for proper mammary development. The data above thus provide further evidence for such conclusions, and demonstrate that Cyclin D1b expression is sufficient to drive proper development of mammary tissue. As a result, additional factors (potentially encompassing both neurological and behavioral aspects) are likely responsible for the observed neonatal lethality of pups born from *Ccnd1*$^{KI/KI}$ mothers as has been demonstrated previously in other GEM models. We thank the reviewer for pointing out these critical experiments and comparisons.

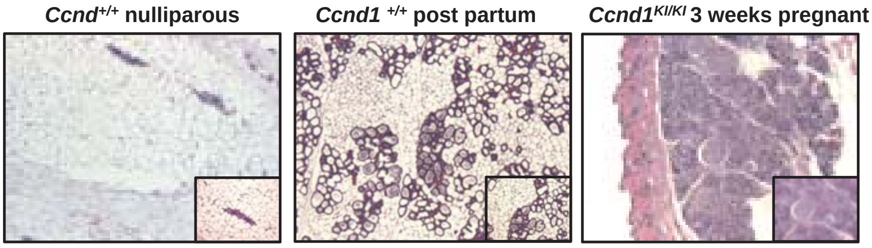

Modified from Revised Figure E1: Mammary tissue of *Ccnd1*$^{KI/KI}$ mice develop normally in response to pregnancy. Mammary tissue from 5 month old *Ccnd1*$^{KI/KI}$ *and* *Ccnd1*$^{+/+}$ mice was harvested from mice within 1 week post partum and analyzed by a board certified veterinary pathologist for glandular development and histo-architecture via H&E staining. Age matched *Ccnd1*$^{+/+}$ females who have never been pregnant serve as a negative control. Images represent 100X magnification with inset taken at 200X.

*11. **Reviewer 2 comment 3:** "Experiments in the latter half of the paper using 3T3s derived from MAFs are interesting, but suffer from a couple of ambiguities. It would therefore be sufficient to characterize primary MAFs for growth rate and serum deprivation response in comparison to WT primary MAFs, and to assess signs of DNA damage, prior to immortalization. It is also important to analyze RB phosphorylation in primary cells, as the persistence of S780 in 3T3s might be the consequence of selection for immortalization. "*

Response: We appreciate the concerns of the reviewer, and have explored characterizing primary non-immortalized cells previously. Unfortunately, cells isolated in this manner undergo rapid cell cycle arrest or death and are often a mix of several cell types prior to immortalization. Consequently, it would be difficult to conclude much from such cell lines. To address these concerns more effectively, the following experiments were completed.

1) Phosphorylation of RB was assessed from several proliferative tissues of both +/+ and KI/KI mice (**Revised Figure E3A)**, where it was found that p-RB S780 in *Ccnd1*$^{KI/KI}$ animals is retained at *Ccnd1*$^{+/+}$ levels. These data demonstrate that proliferative cells which express Cyclin D1b maintain the ability to phosphorylate RB *in vivo* and suggest that the persistence of phosphorylated RB in KI/KI MAF lines was likely not due to the immortalization process.

2) A second independently derived KI/KI MAF line was generated in an identical fashion to the previously described *Ccnd1*$^{+/+}$ and *Ccnd1*$^{KI/KI}$ lines and assessed for markers of genome instability. As now shown in **Revised Figure E4B,** the KI/KI-2 MAF line expresses Cyclin D1b to similar levels of that of the original KI/KI line, and harbors elevated PARP1 activity as compared to *Ccnd1*$^{+/+}$ controls. Furthermore, KI/KI-2 cells are

associated with an enhanced frequency of both p-H2AX and 53BP1 foci, which effectively phenocopy the results generated from the original KI/KI MAF model (**Revised Figure E4A)**. In conjunction with data generated from models of prostate cancer above, these results demonstrate that induction of Cyclin D1b promotes a state of genomic instability across multiple systems, and suggests that a major mechanism though which Cyclin D1b functions in an oncogeneic capacity is mediated through maintenance of this phenotype.

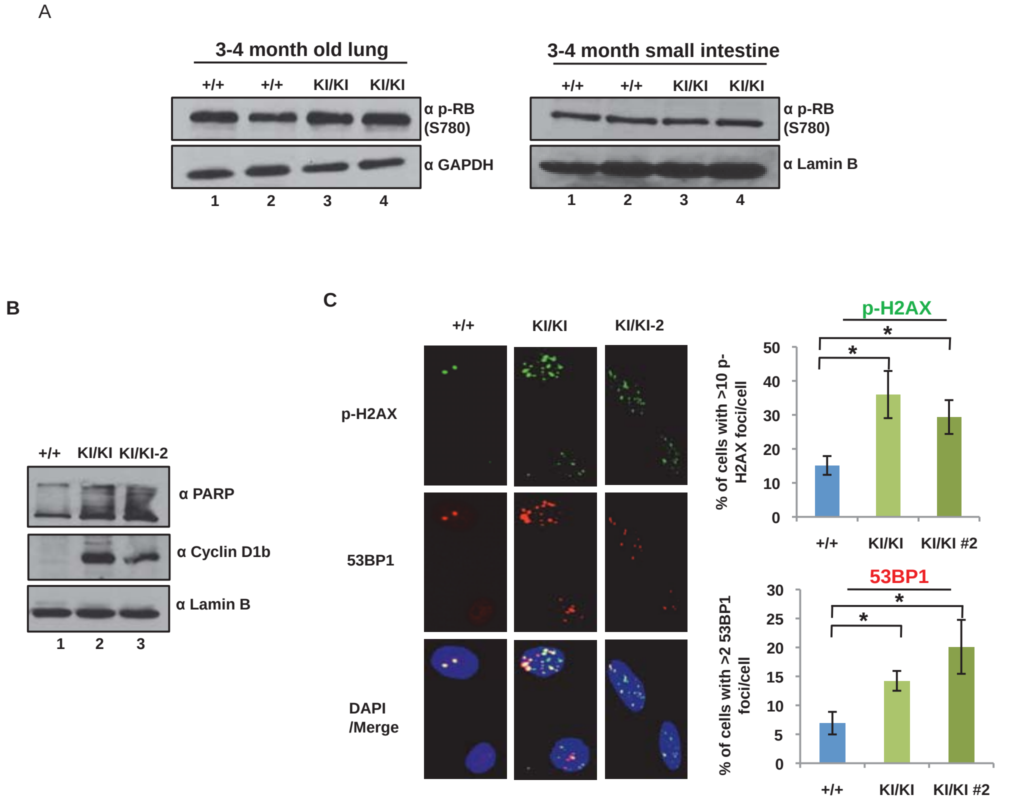

Modified from Revised Figure E3: Cyclin D1b induction is associated with +/+ levels of p-Rb *in vivo* and markers of genomic instability across multiple models *in vitro*. A. Indicated tissues from both *Ccnd1*$^{+/+}$ and *Ccnd1*$^{KI/KI}$ age-matched mice were harvested and assessed for p-RB(S780) via immunoblot. Lamin B and GAPDH serve as controls. B. Indicated (passage matched) cell lines were validated for Cyclin D1b expression and assessed for PARP activity via immunoblot. C. Cell lines in B were fixed and stained for both p-H2AX and 53BP1 foci during log phase growth. The number of foci/cell was determined from three random fields for each cell line in biological triplicate, and is represented as the % of the total cell population with greater than 10 (p-H2AX) or 2 (53BP1) foci/cell. * p<0.05, ** p<0.01

***12. Reviewer 3 comment 1:** "The authors generated MAF (mouse adult fibroblasts) from the KI/KI mice. This seems a strange choice since in most cases, MEFs (mouse embryonic fibroblasts) are generated from mice. The authors need to explain their choice and why MAF are better than MEFs."*

Response: While it is true that murine embryonic fibroblasts are used as models for a multitude of biological processes, murine adult fibroblasts (MAF's) provide an additional resource with which to study both developmental and tumorigenic phenotypes. Indeed many studies have utilized the MAF system to study how manipulation of tumor supressors or oncogenes contribute to transformation both *in vitro* and *in vivo*. Several of these studies also utilized human cancer lines to demonstrate that the effects observed in the MAF system could be recapitulated in human models (also demonstrated in our study- Revised Figure 6). Thus, as we have significant expertise in generating these lines, and as they have shown to be an effective model with which to study tumorigenic phenotypes, we chose to utilize MAF lines in leiu of their embryonic counterpart. (Buorgo et al 2011 Mol Cell), (de Napoles et al 2004 Developmental Cell), (Powers et al 2004, Mol Cancer Research) (Dean et al 2010, Oncogene).

13. Reviewer 3 comment 3: *"The authors study the growth behavior of MAFs and find that they are sensitive to low serum conditions. When they check BrdU incorporation, they find that there is no difference in the KI/KI between 10% and 1% serum but in wild-type MAFs, there is a decrease (as expected). How can the authors explain this contradictory results?"*

Response: This is an issue of misinterpretation, and we apologize for potential confusion in the initial text. The reviewer correctly points out that both +/+ and KI/KI lines demonstrate a reduced proliferative capacity in 1% as compared to full (10% serum) over time. However, the KI/KI lines show an enhanced ability to grow under such conditions (as compared to +/+ control), consistent with a transformed phenotype. To more completely define the acute cell cycle profile under these conditions, both +/+ and KI/KI models were analyzed for BrdU incorporation after 24 hours incubation in 1% serum. Consistent with the growth curve data at this time point, KI/KI lines maintain the capacity to grow with kinetics that mimic full serum, while there is a dramatic and sudden cell cycle arrest in the +/+ control line. As these experiments were designed to define the immediate response to 1% serum, extended time points were not included in this study. However, we anticipate that by 48 hours there would be a reduction in the KI/KI line consistent with the slightly lower proliferative rate observed in the growth curve described above. We again apologize for the confusion and have rigorously modified the text and figure legend in the revised manuscript to clearly articulate this important point.

14. Reviewer 3 comment 5: *"The authors treat MAFs with 5Gy IR and check BrdU incorporation in Fig.6. It seems that the decrease of BrdU incorporation in both wild-type and KI/KI MAFs is quite low at 5Gy IR. How can the authors explain this since these results differ completely what has been published before."*

Response: We apologize for any confusion and have now modified the text to clearly address these points. Both the BrdU incorporation assays as well as the β-galactosidase measurements were conducted 48 hours post treatment. As such, the cells had recovered from the initial insult. These time points were chosen so as to define the ability of both the +/+ and KI/KI lines to recover after DNA damage. However, the reviewer appears to be suggesting that inclusion of an earlier time point may also be of benefit, so as assess relative checkpoint in response to IR. As such, +/+ and

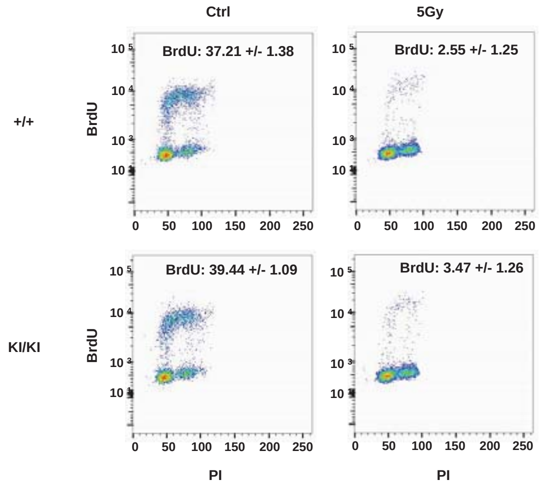

Both +/+ and KI/KI MAF cell lines display rapid cell cycle exit 2 hours post treatment with IR. Indicated cell lines were growth in full serum for 24 hours and then treated with or without 5Gy IR. 1 Hour post treatment, cells were treated with BrdU for 60 minutes, and then harvested for flow cytometry and BrdU incorporation analyses. Data represents the mean % of BrdU positive cells from three biological replicates for each cell line and condition.

KI/KI MAF models were treated with 5Gy IR and assayed for BrdU incorporation 2 hours post treatment, to define the checkpoint response in the presence and absence of Cyclin D1b. As is

shown below both +/+ and KI/KI lines respond effectively to treatment, rapidly exiting the cell cycle and limiting BrdU incorporation. We thank the reviewer for raising this important issue.

2nd Editorial Decision 21 January 2015

Thank you for the submission of your revised manuscript to EMBO Molecular Medicine. We have now received the enclosed reports from the Reviewers that were asked to re-assess it. As you will see the Reviewers are now globally supportive although there remain a few concerns mainly raised by Reviewer 3.

Briefly, Reviewer 1 suggests that you avoid describing CycD1b as a "driver". I agree with his/her argument and request.

Reviewer 2 is quite critical of the use of MAFs rather than MEFs and would like you to comment on this in the manuscript. S/he is also, similarly to Reviewer 1, concerned about the definition of CycD1b as an oncogene and requires clarification on the description of the cross breeding experiments. This Reviewer also notes an issue with the IP experiment depicted in figure E3B, which requires your action. Finally I should mention that I agree with this Reviewer that the manuscript would benefit from a leaner format, although at this stage I would advise against substantial changes.

I am prepared to make an Editorial decision on your final, revised version, provided the issues raised are dealt with as requested. When you do submit, please upload a copy of your manuscript with the changes clearly marked (in addition to the final version).

In the likely event of acceptance, you will be asked to fulfill a number of editorial requirements as listed below. I suggest that you provide the following information and amendments requested directly with the next, final version of your manuscript:

1) As per our Author Guidelines, the description of all reported data that includes statistical testing must state the name of the statistical test used to generate error bars and P values, the number (n) of independent experiments underlying each data point (not replicate measures of one sample), and the actual P value for each test (not merely 'significant' or 'P < 0.05').
2) We are now encouraging the publication of source data, particularly for electrophoretic gels and blots, with the aim of making primary data more accessible and transparent to the reader. Would you be willing to provide a PDF file per figure that contains the original, uncropped and unprocessed scans of all or at least the key gels used in the manuscript? The PDF files should be labeled with the appropriate figure/panel number, and should have molecular weight markers; further annotation may be useful but is not essential. The PDF files will be published online with the article as supplementary "Source Data" files. If you have any questions regarding this just contact me.
3) As per our guidelines, the manuscript must include a statement in the Materials and Methods identifying the institutional and/or licensing committee approving the experiments, including any relevant details (like how many animals were used, of which gender, at what age, which strains, if genetically modified, on which background, housing details, etc). We encourage authors to follow the ARRIVE guidelines for reporting studies involving animals. Please see the EQUATOR website for details: http://www.equator-network.org/reporting-guidelines/improving-bioscience-research-reporting-the-arrive-guidelines-for-reporting-animal-research/. Although I acknowledge that youDecison do provide most information, please complete as necessary.
4) Every published paper now includes a 'Synopsis' to further enhance discoverability. Synopses are displayed on the journal webpage and are freely accessible to all readers. They include a short standfirst (to be written by the Editor) as well as 2-5 one-sentence bullet points that summarise the paper (to be written by the author). Therefore, please provide the short list of bullet points that summarise the key NEW findings. The bullet points should be designed to be complementary to the abstract - i.e. not repeat the same text. We encourage inclusion of key acronyms and quantitative information. Please use the passive voice. Please attach these in a separate file or send them by email, we will incorporate them accordingly.
5) Please upload separate individual files for the main text and each figure. The supplemental information must also be uploaded separately but in this case all material may be included in a

single file.

I look forward to seeing a revised form of your manuscript soon, and possibly no later than two weeks from now.

***** Reviewer's comments *****

Referee #1 (Remarks):

The revised manuscript by Augello and co-workers has addressed all my previous requests to satisfaction. I think that this version is significantly improved and provides interesting mechanistic details of CycD1b function.
My only request now is that I feel that the term "driver" used to describe the oncogenic function of CycD1b may be an overstatement, at least when referring to its in vivo function. The low penetrance and long latency period required for the appearance of spontaneous tumors in vivo in the KI/KI mice together with the in vitro data suggest that CycD1b may be an oncogenic hit that requires additional alterations to trigger tumor onset. Thus the term "driver" (Discussion, page 22 1st paragraph) may be confusing.

Referee #2 (Comments on Novelty/Model System):

This revised study uses a unique and appropriate mouse model system and tissues and cells derived therefrom to carefully characterize an important tumor-promoting variant of cyclin D1, called cyclin D1b. The funciton of this isoform as an oncogene is likely to significantly inform use of cdk4/6 inhibitors likely to be approved for use in many tumor types, and will also stimulate novel thinking about other methods of killing cyclin D1b expressing tumors cells.

Referee #2 (Remarks):

The authors have convincingly and thoroughly addressed my concerns.

Referee #3 (Remarks):

The authors have invested a lot of work in revising their manuscript and have clearly improved it. The fundamental message has not changed and therefore this manuscript is not changing how we think about Cyclin D1.
There are a number of minor issues that need to be addressed before this manuscript can be published:
1. The choice of MAFs is unfortunate given that the majority of research groups use MEFs. This makes comparisons to Cyclin D1-/- MEFs almost impossible since it is a different cell type - in other words, the authors shoot themselves into the foot! They need to include a specific statement about the differences of MAF and MEF and why they used MAFs (unfortunately).
2. "physiological levels of Cyclin D1b are sufficient to promote tumorigenesis, and provides evidence to support its role as a bona fide oncogene." This statement is not correct since it is valid only in the context of immortalized MAFs, which contain additional mutations (otherwise they would not be immortal).
3. "Crosses between Ccnd1+/- mice (>20 mating pairs across multiple generations) revealed that Ccnd1KI/KI mice are born in typical Mendelian ratios" I believe this is sentence is wrong or otherwise I don't understand the genetics behind this cross.
4. E3B, lane 6: The CDK4 IP did not work in comparison to lane 2. This should be repeated.

5. There are problems with references throughout the manuscript.
6. The manuscript is on the long side and would benefit from shortening.

2nd Revision - authors' response 06 February 2015

Thank you for considering our manuscript for revision.

We are pleased that the revised study was well received. As requested, we have addressed the remaining minor textual concerns. Below please find a detailed response to each of the concerns outlining how each point was addressed in the revised manuscript. We look forward to publication in EMBO Molecular Medicine.

Point-by-point-response:

1. Comment 1: We noted that a few manuscript and supplementary figure micrographs are missing size bars or magnification information. Please make sure ALL micrographs have such information.

Response: We have gone through both the text as well as the figures themselves and have modified either the figure legends or figure to include magnification criteria for each micrograph.

2. Comment 2: Thank you for providing a list of acronyms. This is not required, Instead, please make sure they are directly defined in the manuscript upon the first instance of use.

Response: We have gone through the text and ensured that the first instance of each acronym is appropriately defined.

3. I noticed and approve the changes you made in the manuscript as a consequence of the Reviewers' requests. However, it would appear that one of the changes your declare in your response to Reviewer 1 comment 1, namely "These data are the first to demonstrate that physiological levels of Cyclin D1b are sufficient to promote tumorigenesis in immortalized cells, and provides evidence to support its role as an oncogene." does not reflect the actual manuscript which instead currently reads "These data are the first to demonstrate that physiological levels of Cyclin D1b are sufficient to promote tumorigenesis murine cell models, and provides evidence to support its role as an oncogene." Please amend the manuscript to reflect the NEW version as per your rebuttal letter.

Response: We apologize for this oversight, and have corrected the text as requested.
